# *FGFR3* mutation increases bladder tumourigenesis by suppressing acute inflammation

**DOI:** 10.1002/path.5143

**Published:** 2018-09-19

**Authors:** Mona Foth, Nur Faezah Binti Ismail, Jeng Sum Charmaine Kung, Darren Tomlinson, Margaret A Knowles, Pontus Eriksson, Gottfrid Sjödahl, Jonathan M Salmond, Owen J Sansom, Tomoko Iwata

**Affiliations:** ^1^ School of Medicine, Dentistry and Nursing, College of Medical, Veterinary and Life Sciences University of Glasgow Glasgow UK; ^2^ Cancer Research UK Beatson Institute Glasgow UK; ^3^ Leeds Institute of Cancer and Pathology St James's University Hospital Leeds UK; ^4^ Division of Oncology and Pathology, Department of Clinical Sciences Lund University Lund Sweden; ^5^ Division of Urological Research, Department of Translational Medicine Lund University, Skåne University Hospital Malmö Sweden; ^6^ Department of Pathology Queen Elizabeth University Hospital Glasgow UK; ^7^ Institute of Cancer Sciences, College of Medical, Veterinary and Life Sciences University of Glasgow UK

**Keywords:** transitional cell carcinoma, transgenic mouse model, fibroblast growth factors, cancer immunology, neutrophils, inflammation, comparative pathology

## Abstract

Recent studies of muscle‐invasive bladder cancer show that *FGFR3* mutations are generally found in a luminal papillary tumour subtype that is characterised by better survival than other molecular subtypes. To better understand the role of FGFR3 in invasive bladder cancer, we examined the process of tumour development induced by the tobacco carcinogen OH‐BBN in genetically engineered models that express mutationally activated FGFR3 S249C or FGFR3 K644E in the urothelium. Both occurrence and progression of OH‐BBN‐driven tumours were increased in the presence of an S249C mutation compared to wild‐type control mice. Interestingly, at an early tumour initiation stage, the acute inflammatory response in OH‐BBN‐treated bladders was suppressed in the presence of an S249C mutation. However, at later stages of tumour progression, increased inflammation was observed in S249C tumours, long after the carcinogen administration had ceased. Early‐phase neutrophil depletion using an anti‐Ly6G monoclonal antibody resulted in an increased neutrophil‐to‐lymphocyte ratio at later stages of pathogenesis, indicative of enhanced tumour pathogenesis, which supports the hypothesis that suppression of acute inflammation could play a causative role. Statistical analyses of correlation showed that while initial bladder phenotypes in morphology and inflammation were FGFR3‐dependent, increased levels of inflammation were associated with tumour progression at the later stage. This study provides a novel insight into the tumour‐promoting effect of *FGFR3* mutations via regulation of inflammation at the pre‐tumour stage in the bladder. Copyright © 2018 Pathological Society of Great Britain and Ireland. Published by John Wiley & Sons, Ltd.

## Introduction

Bladder cancer is the tenth most common cancer type, particularly in ageing men (Cancer Research UK, http://www.cancerresearchuk.org/about‐cancer/bladder‐cancer/about; accessed on 16 July 2018). The majority of bladder cancers are urothelial cell carcinomas (90%), followed by squamous cell carcinomas. Smoking is a major risk factor for bladder cancer. The majority of urothelial cell carcinomas at diagnosis are non‐muscle‐invasive (NMIBC) (70%), the remainder showing muscle invasion. Muscle‐invasive bladder cancer (MIBC) without metastasis is managed by neoadjuvant chemotherapy followed by radical cystectomy 1. However, the recurrence rate is high, leading to local (10–15%) and distant (50%) metastasis.

Identifying effective therapies has been a challenge for bladder cancer clinically and pre‐clinically, owing to a lack of full understanding of disease mechanisms 2. However, recent molecular analyses of large numbers of MIBCs have defined several molecular subtypes and have identified a range of potential therapeutic targets 3, 4, 5, 6, 7. Abnormal immune regulation promotes tumour progression in many cancer types and could be an effective target for therapy 8. Indeed, bacillus Calmette‐Guérin (BCG) immunotherapy is an effective adjuvant therapy for high‐risk NMIBC that reduces disease recurrence and progression, and is offered as standard therapy 1. More recently, clinical trials of inhibitors of immune checkpoint proteins, such as programmed cell death protein 1 (PD‐1) and PD ligand 1 (PD‐L1), have shown success in advanced bladder cancer in terms of response rate and durability 2, 9. Nevertheless, reliable predictive biomarkers are lacking, and the role of acute and chronic inflammation and tumour immunity is still poorly understood in bladder cancer.

*Fibroblast growth factor receptor 3* (*FGFR3*) mutation and overexpression are common in bladder cancer 10, 11, 12. According to the recent molecular classification of MIBC, tumours with *FGFR3* mutation and overexpression are associated with urothelial‐like or luminal papillary tumour subgroups that are characterised by better survival than other molecular subtypes 7, 13. An activating point mutation in *FGFR3* ^*C746C > G*^, encoding the FGFR3 S249C oncoprotein, accounts for 48–71% of all *FGFR3* mutations in non‐invasive urothelial cell carcinoma 14, 15. S249C affects the linker region between the extracellular immunoglobulin‐like domains Ig2 and Ig3, which is important for the binding of FGF ligands. S249C triggers kinase activation through receptor dimerisation as a result of disulphide bond formation in a completely ligand‐independent manner 16. In contrast, a lysine‐to‐glutamic acid substitution, K650E, in the kinase domain of FGFR3, found in a small number of bladder tumours (∼1% of all mutations), exaggerates ligand‐dependent kinase activation. Overexpression of wild‐type FGFR3 receptor is found in 42% of muscle‐invasive tumours 14. An oncogenic fusion event of FGFR3 with the transforming acidic coiled‐coil containing protein 3 (FGFR3–TACC3), leading to constitutive activation of FGFR3, is also found in bladder cancer 7, 17, 18.

FGFR3 is one of four tyrosine kinase receptors for FGFs 15, 19. *In vitro* studies have provided evidence that mutational activation of FGFR3 through S249C or K644E can modestly increase cell proliferation and reduce apoptosis, and that various FGFR inhibitors are effective in its functional suppression 19. Current clinical trials are based on proof‐of‐principle studies in cell lines and xenograft models 20, 21, 22, 23, 24. Phase II clinical trials of dovitinib, a multi‐targeted RTK inhibitor that prevents phosphorylation of FGFR3, showed limited activity in advanced bladder cancer 25 and in BCG‐unresponsive bladder cancer with mutations or overexpression of FGFR3 26. In contrast, a phase I trial of BGJ398 showed anti‐tumour activity in *FGFR3*‐mutated advanced bladder cancer after failure of platinum‐based chemotherapy 27. A phase I trial using an intermittent dosing schedule of the pan‐FGFR3 inhibitor JNJ‐42756493 on patients with advanced bladder cancer with confirmed FGFR alterations 28 and a case report for phase I AZD4547, a selective FGFR inhibitor targeting FGFR1/2/3 29, also showed promising results.

A better understanding of the role of *FGFR3* mutations in tumour pathogenesis and progression will help in interpreting trial outcomes and allow further stratifications. The use of *in vivo* models closely reflecting the disease conditions would increase robustness and confidence in translation of pre‐clinical findings to trials. Previously, we showed in a mouse model of spontaneous tumour formation that murine Fgfr3 K644E (equivalent to human K650E) in combination with Pten loss was able to induce morphological changes in the urothelium with cellular characteristics indicative of abnormal differentiation 30, 31. One of the most well‐studied bladder carcinogens in mice is *N*‐butyl‐*N*‐(4‐hydroxybutyl)‐nitrosamine (OH‐BBN), derived from tobacco smoke 32. OH‐BBN‐induced tumours are of a highly invasive nature and often show a mixed histology with characteristics of both urothelial cell carcinoma and squamous cell differentiation. Similarities in the histopathology and pathogenesis between the OH‐BBN model and muscle‐invasive bladder tumours in humans have been well established 33, 34.

In this study, we have generated a novel transgenic mouse line that expresses FGFR3 S249C in the urothelium and have compared the effects of OH‐BBN with a wild‐type control, as well as with the previously reported *Fgfr3 K644E* model 30, 31. Furthermore, by neutrophil depletion, we have tested the hypothesis that impairment of acute inflammatory response at an early tumour initiation stage could promote tumour development.

## Materials and methods

### Mice

Generation of *Tg*(*UroII‐hFGFR3IIIbS249C*) (‘*FGFR3* ^*S249C*^’) is described in the supplementary material, Supplementary materials and methods. *UroIICre Fgfr3* ^*+/K644E*^ mice were generated as previously described 31. The wild‐type mice were C57Bl/6 (Charles River, Tranent, UK). The genetic background was C57Bl/6 in all cohorts.

### Carcinogen treatment

Mice were administered 0.05% v/v OH‐BBN (#B0938; TCI UK, Birkenhead, UK) in drinking water three times a week for 10 weeks, starting from 8 to 16 weeks of age, followed by 10 weeks of water. All experiments were performed according to an approved Project Licence under the Home Office Animal (Scientific Procedures) Act 1986.

### Neutrophil depletion

Wild‐type mice were injected (i.p.) with 500 µg of either 1A8 monoclonal antibody (anti‐mLy‐6G; Bioxcell, West Lebanon, NH, USA) or 2A3 isotype control (rat IgG2a; Bioxcell) three times per week for 10 weeks, with concurrent OH‐BBN administration in drinking water. Blood was collected in EDTA‐containing tubes by cardiac puncture following euthanasia. White blood cell populations were analysed using a ProCyte Dx Hematology Analyzer (IDEXX, Westbrook, ME, USA).

### Histology and immunohistochemistry (IHC)

Methods and antibody details are provided in the supplementary material, Supplementary materials and methods.

### Scoring criteria

The number of mice showing the specific criterion was recorded, as assessed on one cross‐section per bladder. On the rare occasion that multiple lesions with different scoring criteria were present within one section, the more severe criterion was assigned. The criteria used for tumour phenotype were: Stage of pathogenesis (minimal changes, urothelial hyperplasia or atypia, dysplastic urothelium or carcinoma *in situ* (CIS), tumour); ‘large’ tumour, tumour size more than 50% of the bladder; ‘small’ tumour, tumour size less than 50% of the bladder; invasiveness (normal basement membrane, ambiguous basement membrane, breakage of basement membrane at multiple sites, stromal invasion, muscle invasion, severe muscle invasion); lobulation of the basement membrane (none, mild or present locally; severe or multiple sites); squamous transformation (none, mild or small area; advanced, fully transformed and often keratinised). Urothelial phenotype at 2 weeks were scored for atypia/dysplasia (minimal changes, atypia, dysplasia). Inflammatory phenotype and neutrophils were scored for thickness of the stroma (normal, thickened, very thickened); angiogenesis in the inner stroma and in the outer stroma (normal, mild increase, notable increase). Neutrophil infiltrations at 2 and 12 weeks were scored in the urothelium, or stroma and muscle, using the criteria <5, 6–20, 21–50, and >50, where section size of the bladders were comparable among samples. Inflammatory phenotype at 20 weeks was scored using the criteria modified from Klintrup *et al*'s method 35 (absent, presence of immune cells sparsely distributed, increase of clustering of immune cells, very prominent inflammatory reaction).

### Analysis of gene expression in TCGA cohorts and statistics

Details are provided in the supplementary material, Supplementary materials and methods. The specific statistical method used is indicated in the figure legends. *p* < 0.05 was considered as statistically significant.

## Results

### Carcinogen‐dependent tumourigenesis was increased in transgenic mice expressing mutationally activated FGFR3 S249C

**Figure 1:**
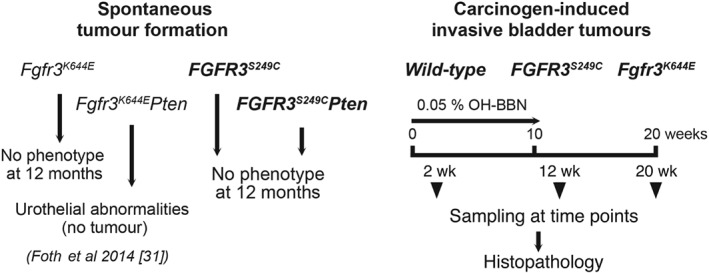
Schematic presentation of the mouse cohorts studied and timeline of carcinogen induction. The role of *FGFR3* mutations was investigated using models of spontaneous tumour formation (left). *FGFR3* mutation by itself, either K644E 30, 31 or S249C (this study), did not lead to urothelial pathogenesis. While double‐mutant mice *Fgfr3* ^*K644E*^ *Pten* resulted in histopathological abnormalities 31, *FGFR3* ^*S249C*^ *Pten* bladders did not show any noticeable histological changes at 12 months. For the carcinogen‐induced model of invasive bladder tumours (right), the tobacco carcinogen OH‐BBN, 0.05% (v/v), was administered to mice in drinking water for the first 10 weeks; tumours were then allowed to develop for a further 10 weeks. The histopathology of the bladders and tumours was examined at 2‐, 12‐, and 20‐week time points.

In order to determine whether an S249C mutation in FGFR3 drives tumour pathogenesis in the bladder, we generated a transgenic mouse line that expresses the human FGFR3 IIIb isoform with an S249C mutation under control of the mouse uroplakin II promoter *Tg*(*UroII‐hFGFR3IIIbS249C*) (‘*FGFR3* ^*S249C*^’). The histological appearance of the *FGFR3* ^*S249C*^ urothelia (*n* = 17) appeared normal at 12 months of age (Figure 1, spontaneous tumour formation; Table 1; and supplementary material, Figure S1). This was similar to observations in mice expressing the isogenic Fgfr3b‐S249C transgene 36, as well as to heterozygous Fgfr3 K644E (*UroIICre Fgfr3* ^*+/K644E*^, ‘*Fgfr3* ^*K644E*^’), which we reported earlier 30, 31, supporting the notion that an *FGFR3* mutation by itself is not able to induce urothelial pathogenesis. Furthermore, bladders of double‐mutant mice with both *FGFR3* ^*S249C*^ and *Pten* loss, ‘*FGFR3* ^*S249C*^ *Pten*’, did not show any noticeable histological abnormalities at 12 months of age (*n* = 12) (Table 1 and supplementary material, Figure S1). This is in contrast to our previous observations in *Fgfr3* ^*K644E*^ *Pten*, which showed histopathological changes indicative of urothelial neoplasia 30, 31.

**Table 1:** Summary of the mouse cohorts

Models of spontaneous tumour formation			
Genotype	Age at time of analysis (months)	Cohort size (*n*) Male (m) Female (f)	Gross observation at time point
Control	10–18	11 (m = 1; f = 10)	None
*FGFR3* ^*S249C*^	6–12	17 (m = 10; f = 7)	Non‐bladder‐related death in *n* = 2 (12%)
*FGFR3* ^*S249C*^ *Pten*	12	12 (m = 6; f = 6)	Non‐bladder‐related death in *n* = 1 (8%)

Carcinogen‐induced bladder cancer model			
Genotype	OH‐BBN treatment (weeks)	Cohort size (*n*)Male (m) Female (f)	Gross observation at time point
Wild type	2	17 (m = 8; f = 9)	None
	12	10 (m = 3; f = 7)	None
	20	47 (m = 20; f = 27)	Tumour (m = 5; f = 1)
*FGFR3* ^*S249C*^	2	15 (m = 10; f = 5)	None
	12	10 (m = 3; f = 7)	None
	20	29 (m = 12; f = 17)	Tumour (m = 3; f = 6)
*FGFR3* ^*K644E*^	2	11 (m = 4; f = 7)	None
	12	8 (m = 3; f = 5)	None
	20	11 (m = 6; f = 5)	Tumour (m = 2; f = 0)

The *Tg*(*UroII‐hFGFR3IIIbS249C*) mouse line (*FGFR3* ^*S249C*^) was generated as described in the supplementary material, Supplementary materials and methods. *UroIICre* 57 and *Pten* ^*flox/flox*^ 58 were inter‐crossed with *FGFR3* ^*S249C*^ to generate *FGFR3* ^*S249C*^ *Pten* ^*flox/flox*^ mice (*FGFR3* ^*S249C*^ *Pten*). The controls were C57Bl/6 (Charles River, UK) (‘wild‐type’) (*n =* 7) and mice with transgenic alleles which do not lead to any phenotype (*n =* 4). The genetic background was C57Bl/6 in all cohorts. For carcinogen induction, mice at 8–16 weeks of age were administered with 0.05% (v/v) OH‐BBN in drinking water for 10 weeks, followed by 10 weeks of normal drinking water. The mice used were wild‐type, *FGFR3* ^*S249C*^, and *UroIICre Fgfr3* ^*+/K644E*^ (*FGFR3* ^*K644E*^) 31.

**Figure 2:**
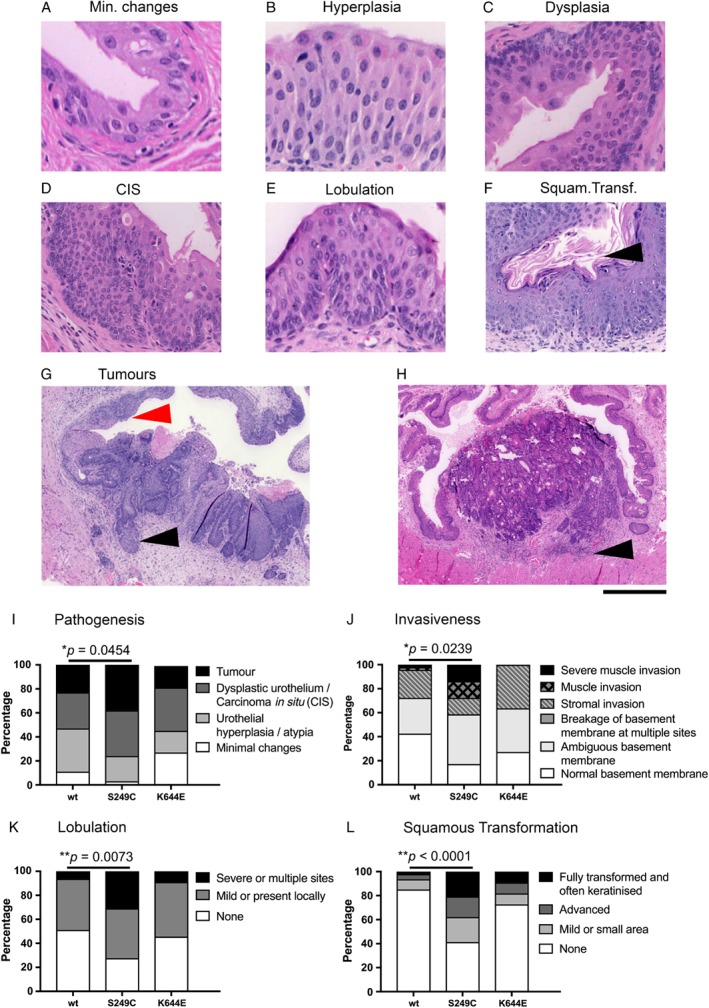
Histopathology of the urothelium and bladder tumours at 20 weeks from the start of carcinogen treatment. Representative images of minimal changes (A), hyperplasia (B), dysplasia (C), and carcinoma *in situ* (CIS) (D). The basement membrane was often lobulated (E) and the urothelium was squamous transformed with keratinised surfaces (arrowhead) (F). An additional example of CIS (red arrowhead) is indicated in the examples of fully developed tumours (G, H) with stromal invasion (black arrowhead) (G). The leading edge of the invading tumour was often infiltrated with inflammatory cells (arrowhead in H). Scale bar represents 50 µm in A, B, E; 70 µm in C; 100 µm in D; 125 µm in F; 500 µm in G; and 700 µm in H. (I–L) The observed phenotype is shown as a percentage of mice that showed the specific phenotypic criterion. (I) Pathogenesis observed in the bladder. (J) Invasiveness of the urothelial and tumour cells. (K) Lobulated appearance of the basement membrane. (L) Squamous differentiation observed in the urothelium and the tumour. In I–L, the number of samples analysed was *n =* 47, 29, and 11, for wild‐type, *FGFR3* ^*S249C*^, and *Fgfr3* ^*K644E*^, respectively (results for males and females combined are shown here). *P* values (Mann–Whitney) are indicated where significant (*p < 0.05 and **p < 0.005).

Next, we used a carcinogen, OH‐BBN, to induce invasive bladder cancer. Since bladder cancer is known to be more frequent in males than in females in humans and in mice 37, 38, we analysed the effects in both genders individually. (The main figures show the combined results from males/females. The results of individual genders are provided in the supplementary figures and are summarised in the supplementary material, Table S1.) At 20 weeks from the start of the carcinogen treatment, mice did not show any overt sign of adverse effects such as haematuria, although tumours in some animals were evident at dissection (Table 1). Metastases were not obvious in any of the cohorts. Subsequently, tumour pathogenesis in the bladder was evaluated histopathologically (Figure 2 and supplementary material, Figure S2). Tumour pathogenesis in *FGFR3* ^*S249C*^ bladders was more advanced in contrast to wild‐type (*p* = 0.0454) (Figure 2I). The invasive nature of the urothelial cells and the tumours was also increased in *FGFR3* ^*S249C*^ (*p* = 0.0239) (Figure 2J). Carcinogen treatment caused the urothelium to show distinct characteristics, including a lobulated basement membrane (Figure 2E) and squamous transformation and keratinisation (Figure 2F). These features were also found to be increased in *FGFR3* ^*S249C*^ compared with wild‐type (*p =* 0.0073 and < 0.0001, respectively) (Figure 2K, L). *Fgfr3* ^*K644E*^ showed two cases of tumour formation (*n* = 2/6 males) which invaded the stroma (Figure 2I).

Overall, the histopathology of carcinogen‐induced tumours was more severe in the presence of an S249C mutation, indicating that both tumour occurrence and progression were enhanced. The phenotype of the *Fgfr3* ^*K644E*^ cohort was less severe than that of the *FGFR3* ^*S249C*^ mice, indicating that the two *FGFR3* mutations are functionally distinct.

### Differential time course of urothelial pathogenesis caused by the two *FGFR3* mutations

**Figure 3:**
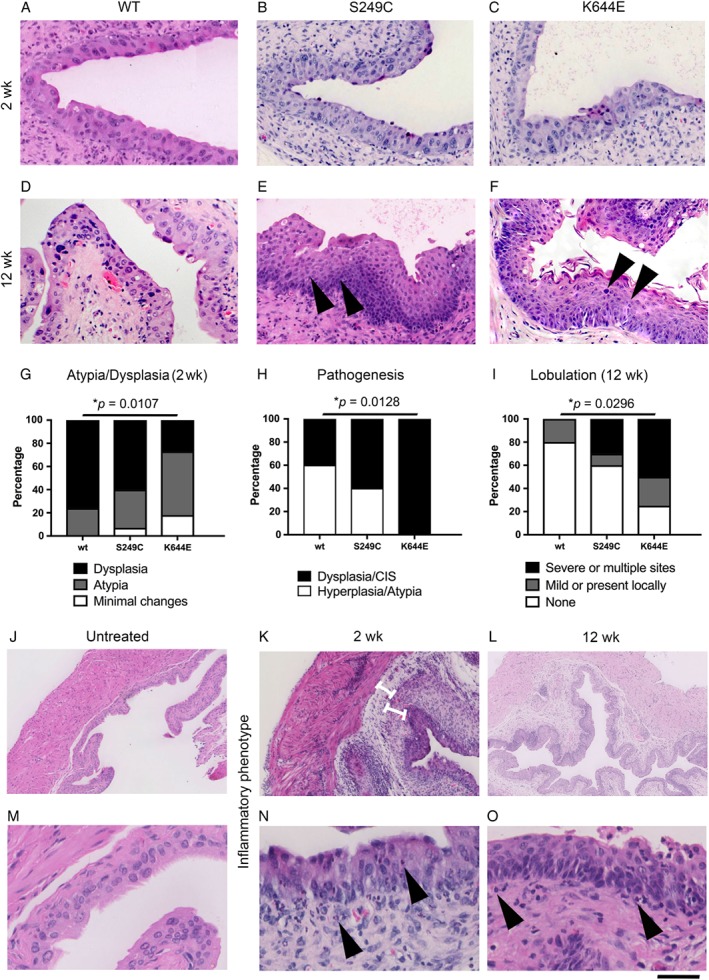
Histopathological and inflammatory phenotype of the bladder at 2 and 12 weeks from the start of carcinogen treatment. Representative H&E images of the urothelium of wild‐type (A, D), *FGFR3* ^*S249C*^ (B, E), and *Fgfr3* ^*K644E*^ mice (C, F) at 2 (A–C) and 12 weeks (D–F) from the start of OH‐BBN treatment. Lobulated basement membrane (arrowheads in E) and squamous transformation (arrowheads in F) were visible at 12 weeks. (G) Presence of atypia and dysplasia in the urothelium at 2 weeks was scored in *n =* 17, 15, and 10 samples of wild‐type, *FGFR3* ^*S249C*^, and *Fgfr3* ^*K644E*^ mice, respectively. Pathogenesis (H) and lobulation of the basement membrane (I) at 12 weeks were scored in *n =* 10, 10, and 8 samples of wild‐type, *FGFR3* ^*S249C*^, and *Fgfr3* ^*K644E*^ mice, respectively. The *Y*‐axis indicates the percentage of mice that showed the specific phenotypic criterion. *P* values (Mann–Whitney) are indicated where significant (*p < 0.05). In the absence of OH‐BBN, no sign of inflammation was present and no neutrophils were observed (J, M). In contrast, 2 weeks of carcinogen treatment caused the bladder to be inflamed with thickened stroma with inner and outer bands (K), with increased presence of neutrophils in the urothelium and in the stroma (arrowheads in N). At 12 weeks from the start of the carcinogen treatment (2 weeks after mice had been returned to the normal drinking water), the bladders showed a mixture of inflamed and normal areas (L). Neutrophils were also observed at 12 weeks (arrowheads) (O). Scale bar represents 100 µm in A–F; 300 µm in J, K; 500 µm in L; and 50 µm in M–O.

We examined the bladder phenotype along the time course of carcinogen treatment (Figure 3, supplementary material, Figure S3 and Table 1). Two weeks of OH‐BBN treatment typically induced atypia and dysplasia and occasional hyperplasia of the urothelium (Figure 3A–C). Contrary to the phenotype at 20 weeks, these characteristics were reduced in the *FGFR3* ^*K644E*^ urothelia (*n =* 10, comparing with wild‐type, *n =* 17, *p* = 0.0107) (Figure 3G).

At 12 weeks from the start of OH‐BBN treatment, which included 10 weeks of OH‐BBN dosing in drinking water followed by 2 weeks without OH‐BBN, the urothelium showed clearer characteristics of tumour pathogenesis, including carcinoma *in situ* (CIS) (Figure 3D–F). Lobulation of the basement membrane and squamous transformation were also apparent (Figure 3E, F). A statistically significant increase in urothelial pathogenesis and lobulation was found in *Fgfr3* ^*K644E*^ (*p =* 0.0128 and 0.0296, respectively) (Figure 3H, I).

Taken together, the increase in tumour pathogenesis became evident as early as 12 weeks from the start of the carcinogen treatment. Unexpectedly, at an early phase of carcinogen induction (2‐week time point), the histopathological changes in the urothelium were suppressed in the presence of the *FGFR3* K644E mutation, indicating differential regulation of urothelial pathogenesis by the two *FGFR3* mutations.

### Neutrophil infiltration was suppressed in *FGFR3* ^*S249C*^ bladders upon carcinogen induction

In an attempt to understand the mechanisms that underlie tumour pathogenesis in *FGFR3* mutant urothelium, we compared the effects of DNA damage caused by OH‐BBN by analysing the levels of γH2AX, p53, p21, and Ki67 (supplementary material, Figure S4), as well as indicators for signalling pathways, including phosphorylation of ERK, AKT, cJUN, and STAT3 (data not shown). No alterations were found in DNA damage response or downstream signalling pathways in the *FGFR3* ^*S249C*^ urothelium.

An acute inflammatory response caused by the administration of OH‐BBN was apparent in the wild‐type bladders at 2 weeks (Figure 3K), in contrast to those not treated by OH‐BBN (Figure 3J). The stroma of the OH‐BBN‐treated bladders was swollen and thickened, accompanied by small blood vessels forming at the inner stroma near the urothelium, and larger vessels were observed closer to the muscle. The overall inflamed appearance of the stroma, scored as stroma thickness and number of blood vessels, was similar between *FGFR3* mutant and wild‐type cohorts (supplementary material, Figure S5A, B). Recruitment of neutrophils to the urothelium, the stroma, and the muscle layer was clearly observed (Figure 3N), while effectively no neutrophils were observed in untreated bladders (Figure 3M). At 12 weeks from the start of OH‐BBN treatment (2 weeks after the last carcinogen treatment), the stroma was typically less swollen than at 2 weeks (Figure 3L); however, neutrophils remained to be frequently observed (Figure 3O).

**Figure 4:**
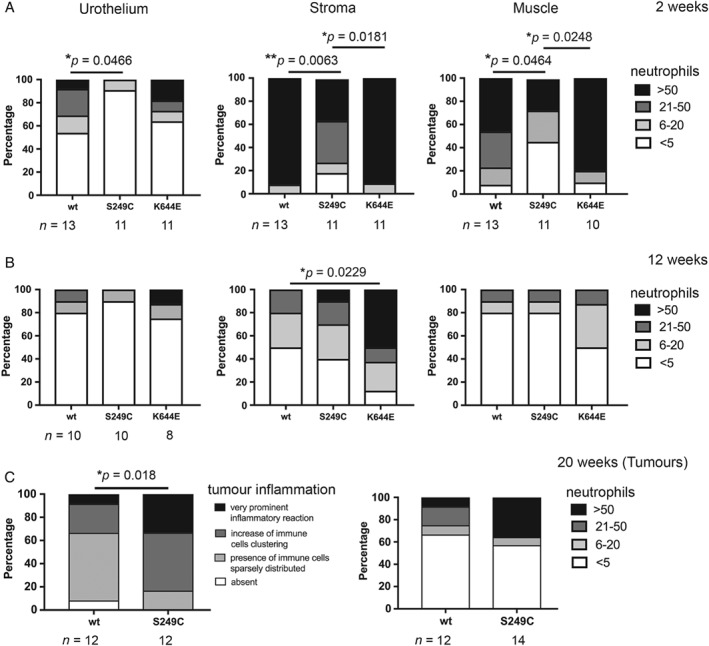
Infiltration of neutrophils in the bladder and bladder tumours at time points of carcinogen treatment. Presence of neutrophils in the urothelium, stroma, and muscle layer of the bladder at 2 weeks (A) and 12 weeks (B) from the start of OH‐BBN treatment. (C) The status of overall inflammation (left) and infiltration of neutrophils (right) in the tumours observed at 20 weeks from the start of OH‐BBN treatment. The *Y*‐axis indicates the percentage of mice that showed the specific phenotypic criterion. The number of samples analysed is indicated below each column. *P* values (Mann–Whitney) are indicated where significant (*p < 0.05 and **p < 0.005).

Interestingly, by scoring the degree of neutrophil infiltration, suppression of neutrophil recruitment to the bladder became apparent in *FGFR3* ^*S249C*^ compared with wild‐type mice at 2 weeks (*p* = 0.0466, 0.0063, and 0.0464 in the urothelium, stroma, and muscle, respectively) (Figure 4A). Infiltration of F4/80^+^ macrophages was also assessed in *FGFR3* ^*S249C*^, indicating that similar suppression may exist in the *FGFR3* ^*S249C*^ urothelium (supplementary material, Figure S5C). At 12 weeks, neutrophil recruitment was no longer suppressed in *FGFR3* ^*S249C*^ (Figure 4B and supplementary material, Figure S6). In contrast to 2 weeks, an increase in neutrophil infiltration was observed in *Fgfr3* ^*K644E*^ stroma compared with wild‐type (*p* = 0.0229) (Figure 4B).

We also investigated the status of tumour inflammation at 20 weeks. Interestingly, tumours were mildly more infiltrated by inflammatory cells in *FGFR3* ^*S249C*^ compared with wild‐type (*p* = 0.018) (Figure 4C). One of two *Fgfr3* ^*K644E*^ tumours was highly infiltrated with neutrophils (score 3), while the other was not (score 0). The level of T‐cell infiltration was similar in *FGFR3* ^*S249C*^ and wild‐type tumours (supplementary material, Figure S7).

These results indicate that the acute inflammatory response to carcinogen treatment, particularly the recruitment of neutrophils to the bladder, was transiently suppressed in the presence of *FGFR3* S249C mutation at the pre‐tumour stage (2 weeks), while at 20 weeks, *FGFR3* ^*S249C*^ bladders were mildly more inflamed than wild‐type.

### Neutrophil depletion during the pre‐tumour stage resulted in increased inflammation

**Figure 5:**
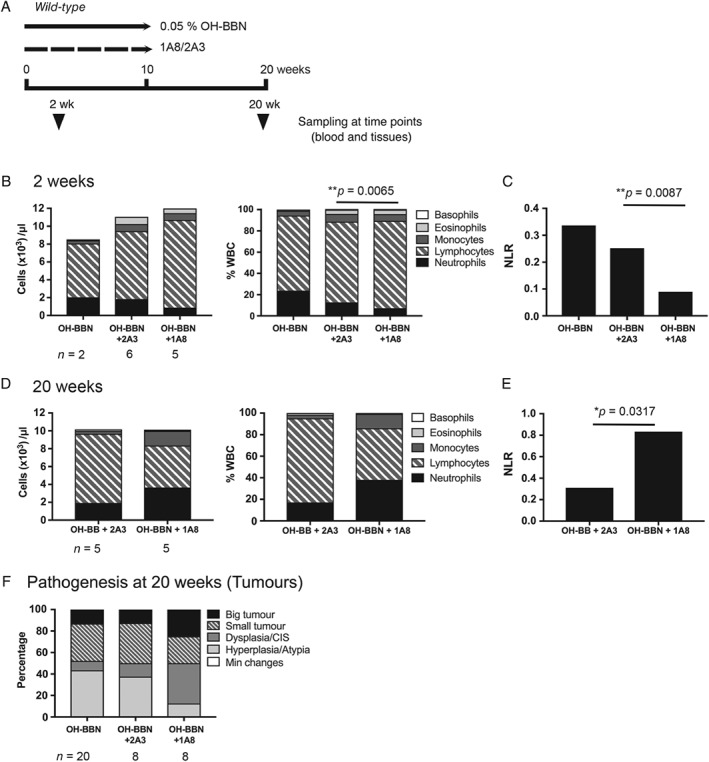
Depletion of Ly‐6G^+^ neutrophils to suppress OH‐BBN‐induced inflammation in the bladder. (A) Schematic presentation of the experiment. A monoclonal antibody against Ly‐6G (1A8) was administered to mice during OH‐BBN treatment for 10 weeks. Clone 2A3 was used as an isotype control. The composition of white blood cells was analysed at 2 weeks (B, C), and at 20 weeks (D, E) from the start of OH‐BBN treatment. Each leukocyte subtype was presented as a proportion within the total white blood cell population (% WBC). (C, E) Neutrophil‐to‐lymphocyte ratio (NLR). (F) Pathogenesis in the bladder at the endpoint, shown as a percentage of mice with the specific phenotypic criterion. The number of samples analysed is indicated below each column. *P* values (Mann–Whitney) are indicated where significant (*p < 0.05 and **p < 0.005).

The early suppression of an acute inflammatory response could result in chronic inflammation later along the process of tumour progression, leading to enhanced tumour pathogenesis in the bladder. To investigate this, neutrophils were depleted using a monoclonal antibody against Ly‐6G^+^ (1A8) along with OH‐BBN treatment in a cohort of wild‐type mice 39 (Figure 5A). At 2 weeks of depletion, 1A8‐treated mice showed significantly lower circulatory neutrophils in comparison with the isotype 2A3‐treated control mice (Figure 5B). The neutrophil‐to‐lymphocyte ratio (NLR) in the blood was similarly reduced upon 1A8 treatment (Figure 5C). At the tissue level, neutrophils were shown to be effectively suppressed in the urothelium of 1A8‐treated mice in comparison to 2A3‐treated mice (supplementary material, Figure S8A).

Next, we sought to determine how neutrophil depletion during the pre‐tumour stage would affect tumour progression. 1A8 was synchronously administered with OH‐BBN for 10 weeks, and the levels of neutrophils were examined at 20 weeks. In contrast to the 2‐week time point, mice had a significantly higher NLR when previously treated with 1A8 (*p* = 0.0317) (Figure 5E). Levels of neutrophil infiltration in the tumour tissue were comparable (supplementary material, Figure S8B). Interestingly, depletion of neutrophils during the pre‐tumour stage indicated a mildly more enhanced severity of tumour pathogenesis at 20 weeks (Figure 5F).

The results of the neutrophil depletion study indicated that the impairment of the acute inflammatory response at the pre‐tumour stage could lead to a later increase in the levels of circulatory immune cells, indicative of enhanced progression of bladder tumours. This supports our hypothesis that the transient suppression of neutrophil recruitment to the bladder in *FGFR3* ^*S249C*^ mice at the pre‐tumour stage could account for increased tumourigenesis.

### Early inflammatory phenotypes are associated with *FGFR3* mutations, while late‐phase inflammation is associated with tumour progression

Changes in the levels of inflammatory infiltrations could be genotype‐dependent (i.e. changes in signalling by *FGFR3* mutant proteins may have regulated the level of inflammation) or, alternatively, phenotype‐dependent (i.e. the inflammatory phenotype may have been caused by the severity of bladder/tumour pathology). In order to address this, we analysed the correlation between genotypes (cohort), phenotypes, and inflammation (supplementary material, Tables S2–S4).

At 2 weeks, dysplasia significantly correlated with the cohort (Spearman's rank test rho: −0.405; *p* = 0.007) (supplementary material, Table S2.1). The Kruskal–Wallis test showed that differences seen among cohorts were statistically significant in dysplasia (*p* = 0.025), neutrophils in the stroma (*p* = 0.003), and in the muscle (*p* = 0.017) (supplementary material, Table S2.4), indicating that both dysplasia and inflammation were genotype‐dependent.

At 12 weeks, cohort‐dependent differences were evident in lobulation (rho: 0.435; *p* = 0.021) and neutrophils in the stroma (rho: 0.435; *p* = 0.022) (supplementary material, Table S3.1). Neutrophils in the stroma were also correlated with bladder phenotypes, including lobulation (rho: 0.671; *p* = 0.00009), and squamous transformation (rho: 0.508; *p* = 0.006). However, no statistically significant links were observed regarding neutrophils by the Kruskal–Wallis tests, either controlled by genotype (supplementary material, Table S3.4) or by pathogenesis (supplementary material, Table S3.7).

At 20 weeks, a positive correlation was evident in scores that indicate tumour pathogenesis (pathogenesis, invasiveness, lobulation and squamous transformation) and inflammation (overall inflammation in the urothelium, stroma, muscle, and tumours) (supplementary material, Table S4.1). A significant correlation with cohort was seen in squamous transformation (rho: 0.323; *p* = 0.002) (supplementary material, Table S4.1). Scores of inflammation were notably correlated with those of pathogenesis. The Kruskal–Wallis test showed that inflammation in tumours was associated with pathogenesis (supplementary material, Table S4.7) and invasiveness (supplementary material, Table S4.10); however, no association was evident when controlled by cohort (supplementary material, Table S4.4).

Taken together, at an early phase of carcinogen induction, regulation of pathogenesis and inflammatory response was associated with the *FGFR3* genotype, indicating the direct causative effects of the *FGFR3* mutations. Once carcinogen treatment had ceased (12 weeks), inflammation was no longer regulated by *FGFR3* mutations. At 20 weeks, only tumour pathogenesis was associated with the FGFR3 genotype, and inflammation was associated with tumour progression.

In humans, tumours with *FGFR3* mutation are associated with the urothelial‐like or luminal papillary tumour subgroup that is generally characterised by lower levels of lymphocytic infiltration 7, 13, 40, 41. Here, we stratified TCGA data 7 by *FGFR3* mutation status and compared the immune gene expression signature 42. In human MIBC, *FGFR3* mutation did not influence the level of immune signature in any of the urothelial‐like/luminal subtypes (supplementary material, Figure S9A, B). When subtypes were grouped together, human urothelial‐like/luminal tumours with *FGFR3* mutation were less immune‐infiltrated than those with wild‐type status (supplementary material, Figure S9C, D). However, this is due to the prevalence of urothelial‐like A‐progressed (UroA‐prog)/luminal‐papillary subtypes that harbour *FGFR3* mutations more frequently, and these subtypes were less immune‐infiltrated among the group regardless of *FGFR3* mutation status (supplementary material, Figure S9A, B).

## Discussion

**Figure 6:**
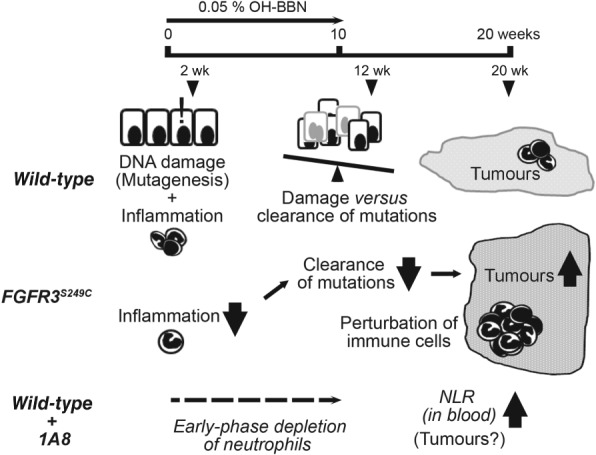
Proposed model of the mechanism underlying increased tumour development in the presence of an *FGFR3* S249C mutation. Carcinogen induces DNA damage in the urothelium as well as an inflammatory response in the bladder that recruits neutrophils to the sites of damage. The balance between DNA damage and clearance of cells that harbour oncogenic mutations by inflammatory response determines the occurrence of the tumour and its pathogenesis. In *FGFR3* ^*S249C*^ mice, reduced inflammatory response at early stages may impair the clearance of DNA‐damaged cells, leading to increased tumour formation and severity at a later stage. Enhanced tumour pathology may accompany perturbation of tumour inflammation. Early‐phase depletion of neutrophils during tumour initiation led to increased circulatory inflammation at a later stage, indicative of enhanced tumour pathogenesis, which supports the notion that suppression of acute inflammation could play a causative role in tumour pathogenesis.

By studying the effects of *FGFR3* mutations using OH‐BBN‐induced, genetically engineered mouse models of invasive bladder cancer, we report three significant findings: firstly, in the presence of mutationally activated FGFR3 S249C, there was an increased number of mice that developed bladder tumours and the tumour phenotype was more advanced (Figure 2). In *FGFR3* ^*S249C*^ mice, tumour cells were more undifferentiated and invasive, with an increase in squamous metaplasia. In the context of the skin, FGFR3 is expressed in keratinocytes, and *FGFR3* mutation was associated with seborrheic keratosis 43. Keratinisation in the mouse model in this study can be interpreted as an advanced feature of tumour progression. Secondly, the inflammatory response was unexpectedly suppressed in the presence of an S249C mutation at an early time of carcinogen induction (Figure 4). Acute inflammation is associated with an anti‐tumour response 8. Reduced clearance of DNA‐damaged cells may have led to overall increased tumour formation in *FGFR3* ^*S249C*^ mice at later stages (proposed in Figure 6). Although involvement of FGF signalling has been reported in inflammatory diseases and in the tumour microenvironment 44, the mechanisms that underlie early suppression of the inflammatory response by S249C‐mutated FGFR3 are currently unknown. FGFR3 is not expressed in neutrophils 45. *FGFR3* mutations are not associated with smoking 46, and are not found in OH‐BBN‐induced tumours in mice 47. Studies on genomic and transcriptional profiles of the tumour samples generated in this study may be useful in order to gain further mechanistic insights into the suppression of acute inflammation and tumourigenesis in the presence of *FGFR3* mutations. Thirdly, established tumours in *FGFR3* ^*S249C*^ mice were mildly more inflamed than wild‐type tumours (Figure 4). This increase in inflammation was mainly associated with overall tumour progression rather than *FGFR3* mutations (supplementary material, Table S4). No significant differences were observed in the levels of T‐cells (supplementary material, Figure S7). Neutrophil depletion at an early phase increased the NLR later at 20 weeks, during the timeframe of tumour establishment and progression, indicative of enhanced tumour pathogenesis (Figure 5). A high circulatory NLR is generally associated with poor prognosis, including in bladder cancer 48, 49, 50, 51. However, the level of neutrophils in the tumours remained similar regardless of early depletion (supplementary material, Figure S8). Therefore, effects of the immune microenvironment on tumour progression are not expected in this model (Figure 5F). Whether suppression of acute inflammatory response leads to tumour inflammation and whether such inflammation reciprocally enhances tumour progression remain unresolved.

*FGFR3* mutations are commonly found in urothelial‐like/luminal papillary tumour subtypes generally associated with a better prognosis and accompanied by low levels of lymphocytic infiltration 7, 13, 40, 41. In the context of PD‐1/PD‐L1 checkpoint blockade therapy, presence of T‐cell populations was shown to be an important indicator for the patients' response, where low levels of CD3^+^ and CD8^+^ T‐cells are associated with a poor outcome 9, 51, 52. It has been reported recently that *FGFR3* mutations are frequently found in non‐CD8^+^ T‐cell‐inflamed MIBC, and it was proposed that the FGFR3 pathway could be targeted to overcome resistance and sensitise tumours to PD‐1/PD‐L1 immunotherapy 40. Our analysis of urothelial‐like/luminary papillary tumour subtypes in the TCGA dataset showed that the expression of immune genes was not affected by the presence or absence of *FGFR3* mutations in each of the different subtypes of the Lund or TCGA classification (supplementary material, Figure S9). This included urothelial‐like B (UroB), a subtype with the worst overall survival, similar to small‐cell/neuroendocrine‐like (Sc/NE‐like) 13. Lower infiltration was indeed associated with *FGFR3* mutation when all subtypes within the urothelial‐like/luminary papillary subtypes were combined; however, this is due to the frequency of lower‐infiltrated subgroups, such as UroA‐Prog/luminal‐papillary subtypes (supplementary material, Figure S9C, D). The lack of a difference in immune gene expression within the tumour subtype in the presence and absence of *FGFR3* mutation in human MIBC could be due to the fact that human studies are based on established tumours, while our study in mouse models investigated the functional effects of FGFR3 along the process of tumour initiation and development, the earlier phases in tumour pathogenesis. It would be interesting to compare the levels of tumour inflammation in OH‐BBN‐induced bladder models with other molecular changes, such as *Tp53*, *Stat3*, and *Sparc* models 53. To facilitate the evaluation, we have previously generated the ‘tumour progression scale’ in phenotyping mouse bladder tumours with an inflammatory phenotype 54.

Examining the individual *FGFR3* mutations, early suppression of neutrophil infiltration was not present in the *Fgfr3* ^*K644E*^ mice (Figure 4). Instead, an increase in pathogenesis was observed at 2 and 12 weeks (Figure 3), and stromal neutrophil infiltration was increased in *Fgfr3* ^*K644E*^ mice (Figure 4B), indicating earlier kinetics in urothelial pathogenesis. Nonetheless, it did not lead to a significant increase in tumourigenesis at the 20‐week endpoint (Figure 2). Therefore, the effects of individual *FGFR3* mutations in regulating neutrophils and tumour pathogenesis are distinct and may explain the low frequency of kinase domain mutations in human bladder neoplasia. Mechanistically, the way that these two mutations regulate the receptor protein kinase function and downstream signalling could be different 15, 16. For example, S249C leads to phosphorylation of PLCγ1, while the equivalent kinase domain mutation K652E does not 55. Cell–cell and cell–matrix adhesion was also indicated to be differently regulated in cells expressing S249C and K652E 56. Effects of gender in bladder cancer epidemiology and the underlying mechanism have been well discussed 37, 38. The results of male/female combined analyses were consistent with those done individually (summarised in the supplementary material, Table S1). However, the small number of mice used may have masked any effects.

In summary, our study showed that the increased tumour progression could be initiated by the effects of *FGFR3* mutations in regulating an acute inflammatory response, and that immune cells are perturbed in the tumour as a consequence. Clinically, it would be beneficial to explore FGFR3 inhibition together with the concurrent immune modulators, such as BCG, as a potential treatment strategy for FGFR3‐mutated or ‐overexpressing bladder cancer at an early stage.

## Author contributions statement

MF, NFBI, JSCK, and TI performed experiments and analysed the results. DT and MK designed and generated the *FGFR3* ^*S249C*^ transgene vector. PE and GS performed bioinformatic evaluation of the role of *FGFR3* mutation in Lund/TCGA cohorts. JMS advised on histopathology. MF, NFBI, JSCK, and TI wrote the manuscript. OJS and TI supervised the overall project. All authors edited the draft manuscript.

**SUPPLEMENTARY MATERIAL ONLINE**

**Supplementary materials and methods**

**Figure S1.** Urothelial appearance of mice with *FGFR3* S249C mutation at 12 months

**Figure S2.** Histopathology of the urothelium and the bladder tumours at 20 weeks from the start of the carcinogen treatment

**Figure S3.** Histopathology of the bladder at 2 and 12 weeks from the start of the carcinogen treatment

**Figure S4.** Response to DNA‐damaging effects of OH‐BBN treatment

**Figure S5.** Inflammatory characteristics of the bladder at 2 weeks of carcinogen treatment

**Figure S6.** Presence of the neutrophils in the bladder at 12 weeks of carcinogen treatment

**Figure S7.** Inflammatory phenotype of the bladder at 20 weeks from the start of application of carcinogen

**Figure S8.** Presence of neutrophils in the bladder tissues upon neutrophil depletion

**Figure S9.** Immune signature expression in TCGA datasets comparing *FGFR3* mutant and wild‐type stratified by Lund or TCGA molecular subtype

**Table S1.** Summary of phenotype compared by gender

**Table S2.** Correlation of inflammatory phenotype by cohort and by bladder phenotype at 2 weeks

**Table S3.** Correlation of inflammatory phenotype by cohort and by bladder phenotype at 12 weeks

**Table S4.** Correlation of inflammatory phenotype by cohort and by tumour phenotype at 20 weeks

## Supporting information


**Appendix S1.** Supplementary materials and methods

**Figure S1. Urothelial appearance of mice with *FGFR3 S249C* mutation at 12 months.** Representative sections of *Wildtype, FGFR3S249C* and *FGFR3S249CPten* stained by H&E (**A‐C**), Ki67 (**D‐F**) and anti‐FGFR3 (B‐9) antibody (**G‐I**). (**D‐F**) Few cells within the urothelium showed positivity with cell proliferation marker Ki67. Mainly stromal cells were positive. No significant difference in Ki67 staining was observed between *Wildtype* Control, *FGFR3S249C* or *FGFR3S249CPten* mice. (**G‐I**) Similar levels and patterns of FGFR3 expression were observed in the *Wildtype, FGFR3S249C* and *FGFR3S249CPten* urothelium. Immunohistochemistry was performed in n=3 samples per genotype. Scale bar represents 100 µm (**A‐I**).
**Figure S2. Histopathology of the urothelium and the bladder tumours at 20 weeks from the start of the carcinogen treatment.** The phenotype was analysed in male and female cohorts separately. Frequency of the observed phenotype was shown as percentage within the samples analysed. **(A)** Pathogenesis observed in the bladder. 23.4% of *Wildtype* mice (n=11/47) formed well‐recognisable tumours of an invasive nature (Fig 2I). When male and female mice were analysed separately, tumours had occurred in 40.0% (n=8/20) and 11.1% (n=3/27), respectively. This is in concordance with the increased frequency of bladder cancer in males. (**B**) Invasiveness of the urothelial and tumour cells. (**C**) Lobulated appearance of basement membrane. (**D**) Squamous differentiation observed in the urothelium and the tumour. Number of samples analysed for each phenotype is indicated below each column. The *p*‐values (Mann‐Whitney) are indicated above the columns, when significant (* <0.05 and ** < 0.005).
**Figure S3. Histopathology of the bladder at 2 and 12 weeks from the start of the carcinogen treatment.** Phenotype was analysed in both genders (**M/F**), male and female cohorts at 2 weeks (**A**) and 12 weeks (**B‐D**). The frequency of each phenotypic criterion was shown as percentage within mice analysed. Presence of atypia and dysplasia (**A**) in the urothelium at 2 weeks, (**B**) Pathogenesis observed in the bladder, (**C**) lobulated appearance of the basement membrane and (**D**) squamous differentiation observed in the urothelium at 12 weeks were scored. Results of the analysis of M/F combined for atypia/dysplasia at 2 weeks, pathogenesis and lobulation at 12 weeks are presented in Figure 3. Number of samples analysed is indicated below panels. The *p*‐values (Mann‐Whitney) are indicated where significant (* <0.05 and ** < 0.005).
**Figure S4. Response to DNA‐damaging effects of OH‐BBN treatment.** The absence of specific differences in the pattern of γH2aX phosphorylation, expression of p53, p21, proliferation between *Wildtype* and *FGFR3S249C*, excludes dysregulation of the Phospho‐γH2aX‐p53 axis as the potential cause of malignancy. IHC was performed in the minimum of n=5 samples per cohort at each time point. Representative sections of Wildtype and *FGFR3S249C* bladders without OH‐BBN treatment (**A, B, G, L, Q, R**), at 2 weeks (**C, D, H, I, M, N, S, T**) and 12 weeks from the start of the OH‐BBN treatment **(E, F, J, K, O, P, U, V)** stained by Phospho‐γH2aX **(**A‐F), p53 (**G‐K**), p21 (**L‐P**) and Ki67 (**Q‐V**). When untreated with OH‐BBN, *FGFR3S249C* bladders showed similar staining pattern to controls, including p53 and p21 (**G, L**). In the absence of carcinogen treatment, γH2aX phosphorylation was scarcely seen, indicating that the DNA damage was minimal in both cohorts (**A, B**). Proliferation of the urothelial cells was similarly low in *Wildtype* and *FGFR3S249C* (**Q, R**), consistent with our earlier observations [30,31]. At 2 weeks of OH‐BBN treatment, increased γH2aX phosphorylation was seen in *Wildtype* and *FGFR3S249C* (**C, D**) compared to untreated urothelia (**A, B**). p53 protein levels were elevated along the basal cell layer as well as in some of the intermediate cells (**H, I**). p21 expression was evident in all urothelial layers (**M, N**). Increased proliferation was typically found along the basal layer of cells (**S, T**). These changes appeared to be according to the expected response to the carcinogen, and were similar between *Wildtype* and *FGFR3S249C* cohorts. At 12 weeks, γH2aX phosphorylation generally remained in all layers of the urothelium in *Wildtype*, as well as in *FGFR3S249C* mice (**E, F**). However, cells that had undergone squamous cell transformation appeared to have lost γH2aX phosphorylation in the basal cell layer (**F**), indicating that cells with DNA damage were cleared from these areas. Expression of p53 was strong in all layers of the urothelium, in particular along the basal cell layer and in some of the intermediate cells (**J, K**), while some urothelial regions lacked p53 expression (**K**). Compared to 2 weeks of OH‐BBN exposure, the expression of p21 was less intense at 12 weeks (**O, P**) and no longer showed any association with regions of high p53 expression as seen at 2 weeks. Proliferation was generally observed along the basal cell layer and often regionally intense (**U, V**). Scale bar represents 100 µm in all panels.
**Figure S5. Inflammatory characteristics of the bladder at 2 weeks of carcinogen treatment.** Phenotype was analysed in both genders (**M/F**), male and female cohorts and shown as percentage within the samples analysed. (**A**) Thickness of the stroma and (**B**) the presence of blood vessels in the stroma closer to the urothelium (inner stroma). No difference was detected in the presence of blood vessels closer to the muscle (the outer stroma) comparing cohorts (data not shown). (**C**) Macrophages in the urothelium were scored using IHC with F4/80. There were no differences in the number of macrophages in the stroma and muscle comparing *Wildtype* and *FGFR3S249C* (data not shown). Presence of neutrophils in the urothelium (**D**), stroma (**E**)**,** and muscle layer (**F**) of the bladder. Number of samples analysed is indicated below panels. Number of samples analysed is indicated below each column. The *p*‐values (Mann‐Whitney) are indicated where significant (* <0.05).
**Figure S6. Presence of the neutrophils in the bladder at 12 weeks of carcinogen treatment.** Presence of neutrophils in the urothelium (**A**), stroma (**B**)**,** and muscle layer (**D**) of the bladder at 12 weeks from the start of OH‐BBN treatment. Phenotype was analysed in male and female cohorts and shown as percentage within the samples analysed. Number of samples analysed is indicated below each column in C. The *p*‐values (Mann‐Whitney) are indicated where significant (* <0.05).
**Figure S7. Inflammatory phenotype of the bladder at 20 weeks from the start of application of carcinogen.** Presence of overall levels of inflammatory infiltrations (**A**) and neutrophils (**B**) in the tumour was analysed in males and females individually. Infiltration of the T‐cell population was analysed using CD3 as a marker in the urothelium (**C**), CIS (**D**) and in tumours (**E**). The Y axis indicates percentage within the samples analysed. Number of samples analysed is indicated below each column. The *p*‐values (Mann‐Whitney) are indicated where significant (* <0.05).
**Figure S8. Presence of neutrophils in the bladder tissues upon neutrophil depletion.** (**A**) Efficiency of neutrophil depletion was evaluated in the bladder tissues at 2 weeks from the start of the carcinogen treatment. (**B**) Infiltration of neutrophils in the tumour were examined at 20 weeks time point. The Y axis indicates percentage within the samples analysed. Number of samples analysed is indicated below each column. Differences were not statistically significant.
**Figure S9. Immune signature expression in the TCGA dataset comparing *FGFR3* mutant and Wildtype stratified by Lund or TCGA molecular subtype.** Immune infiltration signature in Lund Urothelial‐Like subtype (n=170) (**A**), TCGA Luminal subtype (n=246) (**B**), and all tumours grouped by *FGFR3* mutation status in Lund Urothelial‐Like subtype (**C**), and TCGA Luminal subtype **(D**). The *p*‐values (Wilcoxon rank test) are indicated. The expression of immune genes was not affected strongly by the presence or absence of *FGFR3* mutations within the different subtypes of the Lund or TCGA classification (**A, B**). The immune expression did not vary significantly within Urothelial‐like B (UroB) based on *FGFR3* mutational status (*p*=0.83), while UroB as a whole and UroB with *FGFR3* mutations were more infiltrated than Urothelial‐like A‐Progressed (UroA‐Prog) (*p*=0.0067 and 0.01 respectively) (**A**). FGFR3 mutations are uncommon in the Urothelial‐like Infiltrated (Uro‐Inf) and Urothelial‐like C (UroC) groups. The infiltration level in Uro‐Inf and UroC was higher than in UroAProg tumors (*p*= 4.8e‐09 and 8.9e‐09, respectively). Similarly, no difference in infiltration was seen between *FGFR3* mutated and wild‐type tumors of the TCGA Luminal‐Infiltrated group (*p*=0.92) (**B**). TCGA Luminal‐Infiltrated had higher immune infiltration than the Luminal‐Papillary group (*p*<2.2e‐ 16). (**C, D**) When grouped by *FGFR3* mutation status, it appears that *FGFR3* mutated Urotheliallike/ Luminal tumors had moderately lower immune infiltration. However, the lower infiltration seen in the mutated cases is due to an enrichment of mutations in the less infiltrated UroA‐prog and Luminal‐Papillary subtypes, since the mutated cases in **C, D** are not significantly different from *FGFR3* wild‐type UroA‐prog/Luminal‐papillary tumours, as shown in **A, B**.

**Table S1.** Summary of phenotype compared by gender
**Table S2.** Correlation of inflammatory phenotype by cohort and by bladder phenotype at 2 weeks
**Table S3.** Correlation of inflammatory phenotype by cohort and by bladder phenotype at 12 weeks
**Table S4.** Correlation of inflammatory phenotype by cohort and by tumour phenotype at 20 weeks
