# Supplementary material for: FGFR3 mutation increases bladder tumourigenesis by suppressing acute inflammation
Source: J Pathol. 2018 Sep 19;246(3):331–43. doi: 10.1002/path.5143 (PMC6334176; doi:10.1002/path.5143)
Supplement: Supplementary file 1 — Appendix S1. Supplementary materials and methods [file PATH-246-331-s001.doc]

Supplementary materials and methods

Reference numbers refer to the main text reference list

**Generation of transgenic mice**

A mouse *Uroplakin II* (*UroII*) fragment (2.5 kb) and human FGFR3IIIb cDNA mutated at position C764C>G were cloned using SalI and NotI sites into a pGEM vector (Promega, Madison, WI, USA). A poly-A sequence was added 156 bp after the 3' end of the FGFR3 cDNA. The resulting *pGEM-UroII-hFGFR3IIIb-S249C* construct was formed of 10 294 bp and was fully sequenced for confirmation. *FGFR3 S249C* transgenic animals were generated by pronuclear injection of the *pGEM-UroII-hFGFR3IIb-S249C* vector. Two mice from the *Tg*(*UroII-hFGFR3IIIbS249C*) transgenic F2 offspring on a C57/CBA mixed background were further crossed with C57Bl/6 mice. The presence of the *FGFR3 S249C* mutation in *Tg*(*UroII-hFGFR3IIIbS249C*) bladders was confirmed by PCR and sequencing of isolated genomic DNA from mouse tail tips. Subsequently, five mice that tested positively for the *S249C* mutation were bred to C57Bl/6 mice in order to establish a line. Genotyping was performed by Transnetyx, Cordova, TN, USA.

**Histology**

Bladders were gently emptied of urine and placed in 10% neutral buffered formalin for overnight fixation. Fixed bladders were embedded in paraffin, with the bladder dome facing up and the trigone down. Haematoxylin and eosin (H&E) staining was performed on 4-µm-thick paraffin sections.

**Immunohistochemistry (IHC)**

Antigen retrieval was performed with 0.01 m citric acid (pH 6) unless specified otherwise. Samples were incubated in 0.3–3% H_2_O_2_ in distilled water for 20 min. Following blocking with 2.5% normal horse serum (S-2012, ImmPRESS; Vector Labs, Peterborough, UK), incubation with the primary antibody was performed for 1 h at room temperature and then overnight at 4°C, with secondary antibody incubation for 30 min to 2 h at room temperature. Antibody binding was visualised by an ABC Elite Standard kit (Vector Labs; PK-6100) and 3,3'-diaminobenzidine (DAB; K3468; Dako, Stockport, UK), counterstained with haematoxylin. IHC staining was examined in a minimum of *n* = 3 mice per genotype. The antibodies used were FGFR3 (Santa Cruz Biotechnology, Dallas, TX, USA; B-9, sc-13121; 1:100, antigen retrieval at pH 9), Ki67 (Vector Labs, Burlingame, CA, USA; VP-RM04; 1:100), phospho-γH2AX (Cell Signaling, Danvers, MA, USA; #9718; 1:50), p53 (Vector Labs; VP-P956; 1:150), p21 (Santa Cruz Biotechnology; M19; 1:500), F4/80 (Abcam, Cambridge, UK; ab111101; 1:50), and CD3 (Vector Labs; VP-RM01; 1:250). Biotinylated goat secondary antibodies anti-rabbit IgG (BA-1000), anti-rat IgG (BA-9401), and anti-mouse IgG (BA-9200; all Vector Labs) were used. Slides were scanned by a Nanozoomer slide scanner (Hamamastu, Shizuoka, Japan) and analysed by the SlidePath Digital Image Hub (Leica Biosystems, Wetzlar, Germany).

**Statistics**

Prism 7 (GraphPad Inc, La Jolla, CA, USA) and SPSS Statistics Version 19 (IBM, Armonk, NY, USA) programs were used. The Mann–Whitney test was used to evaluate non-parametric distribution of data for individual significance between the genotypes. Spearman’s rank test was used to evaluate relationships between two categorical variables, such as cohort versus hyperplasia. The Kruskal–Wallis test was used to define overall significance of differences (such as between cohorts) across multiple categories (such as phenotypes).

**Gene expression analysis of the TCGA cohorts and statistical analysis**

To investigate the expression levels of immune-related genes in human bladder tumours, we made use of the publicly available TCGA dataset in which molecular subtypes have been determined both by TCGA classification [7] and by the Lund Taxonomy (LundTax [13]). Since *FGFR3* mutations occur primarily in the TCGA luminal-like subtypes and in the LundTax urothelial-like subtypes, we focused our analysis on these subsets. TCGA luminal-like cases were further subdivided into luminal-papillary, luminal, and luminal-infiltrated. The Lund urothelial-like subset was further subdivided into urothelial-like A-progressed (UroA-Prog), urothelial-like B (UroB), urothelial-like C (UroC), and urothelial-like infiltrated (Uro-Infiltrated). TCGA data sets for RNA-seq (*N* = 407) and mutations (*N* = 389) were downloaded and used as described in Marzouka *et al* [13]. In brief, RNA-seq data were downloaded from the Xena hub ([http://xena.ucsc.edu](http://xena.ucsc.edu/)) at UCSC as log_2_, normalized RSEM values which were median-centred prior to analysis. We downloaded the TCGA exome-sequencing data (MAF format) from the Broad Institute of MIT and Harvard (10.7908/C1MW2GGF) for 389 cases and identified the missense substitutions in the coding region of *FGFR3.* The vast majority of the identified mutations were located in the known hot-spots, and presence or absence of mutation was used to determine mutation status in the analyses. The gene expression levels for 141 tumour immune-infiltration genes [42] were used to test variation in tumour immune-signals as a function of molecular subtype and *FGFR3* mutation status. Only the 389 cases for which both RNA-seq and mutation data were available were included in the analyses. Differences between groups were assessed by the Wilcoxon rank-test, and the calculations were performed in R (<http://www.r-project.org/>).
