# Supplementary material for: FGFR3 mutation increases bladder tumourigenesis by suppressing acute inflammation
Source: J Pathol. 2018 Sep 19;246(3):331–43. doi: 10.1002/path.5143 (PMC6334176; doi:10.1002/path.5143)
Supplement: Supplementary file 2 — Figure S1. Urothelial appearance of mice with FGFR3 S249C mutation at 12 months. Representative sections of Wildtype, FGFR3S249C and FGFR3S249CPten stained by H&E (A‐C), Ki67 (D‐F) and anti‐FGFR3 (B‐9) antibody (G‐I). (D‐F) Few cells within the urothelium showed positivity with cell proliferation marker Ki67. Mainly stromal cells were positive. No significant difference in Ki67 staining was observed between Wildtype Control, FGFR3S249C or FGFR3S249CPten mice. (G‐I) Similar levels and patterns of FGFR3 expression were observed in the Wildtype, FGFR3S249C and FGFR3S249CPten urothelium. Immunohistochemistry was performed in n=3 samples per genotype. Scale bar represents 100 µm (A‐I). Figure S2. Histopathology of the urothelium and the bladder tumours at 20 weeks from the start of the carcinogen treatment. The phenotype was analysed in male and female cohorts separately. Frequency of the observed phenotype was shown as percentage within the samples analysed. (A) Pathogenesis observed in the bladder. 23.4% of Wildtype mice (n=11/47) formed well‐recognisable tumours of an invasive nature (Fig 2I). When male and female mice were analysed separately, tumours had occurred in 40.0% (n=8/20) and 11.1% (n=3/27), respectively. This is in concordance with the increased frequency of bladder cancer in males. (B) Invasiveness of the urothelial and tumour cells. (C) Lobulated appearance of basement membrane. (D) Squamous differentiation observed in the urothelium and the tumour. Number of samples analysed for each phenotype is indicated below each column. The p‐values (Mann‐Whitney) are indicated above the columns, when significant (* <0.05 and ** < 0.005). Figure S3. Histopathology of the bladder at 2 and 12 weeks from the start of the carcinogen treatment. Phenotype was analysed in both genders (M/F), male and female cohorts at 2 weeks (A) and 12 weeks (B‐D). The frequency of each phenotypic criterion was shown as percentage within mice analysed. Presence of atypia and dysplasia (A [file PATH-246-331-s002.pdf]

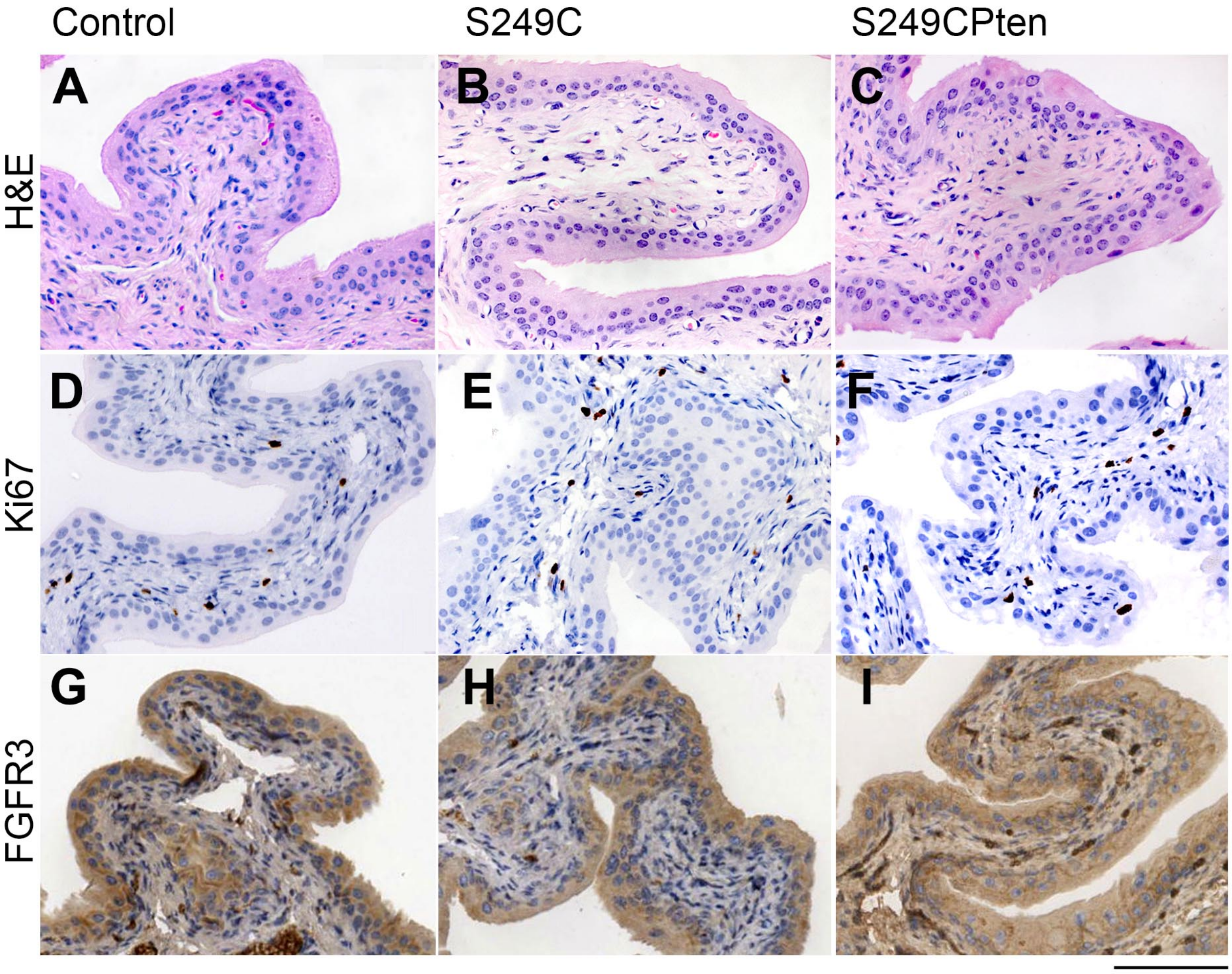

Figure S1. Urothelial appearance of mice with *FGFR3 S249C* mutation at 12 months. Representative sections of *Wildtype, FGFR3*^{S249C} and *FGFR3*^{S249C}*Pten* stained by H&E (**A-C**), Ki67 (**D-F**) and anti-FGFR3 (B-9) antibody (**G-I**). (**D-F**) Few cells within the urothelium showed positivity with cell proliferation marker Ki67. Mainly stromal cells were positive. No significant difference in Ki67 staining was observed between *Wildtype* Control, *FGFR3*^{S249C} or *FGFR3*^{S249C}*Pten* mice. (**G-I**) Similar levels and patterns of FGFR3 expression were observed in the *Wildtype, FGFR3*^{S249C} and *FGFR3*^{S249C}*Pten* urothelium. Immunohistochemistry was performed in n=3 samples per genotype. Scale bar represents 100 µm (**A-I**).

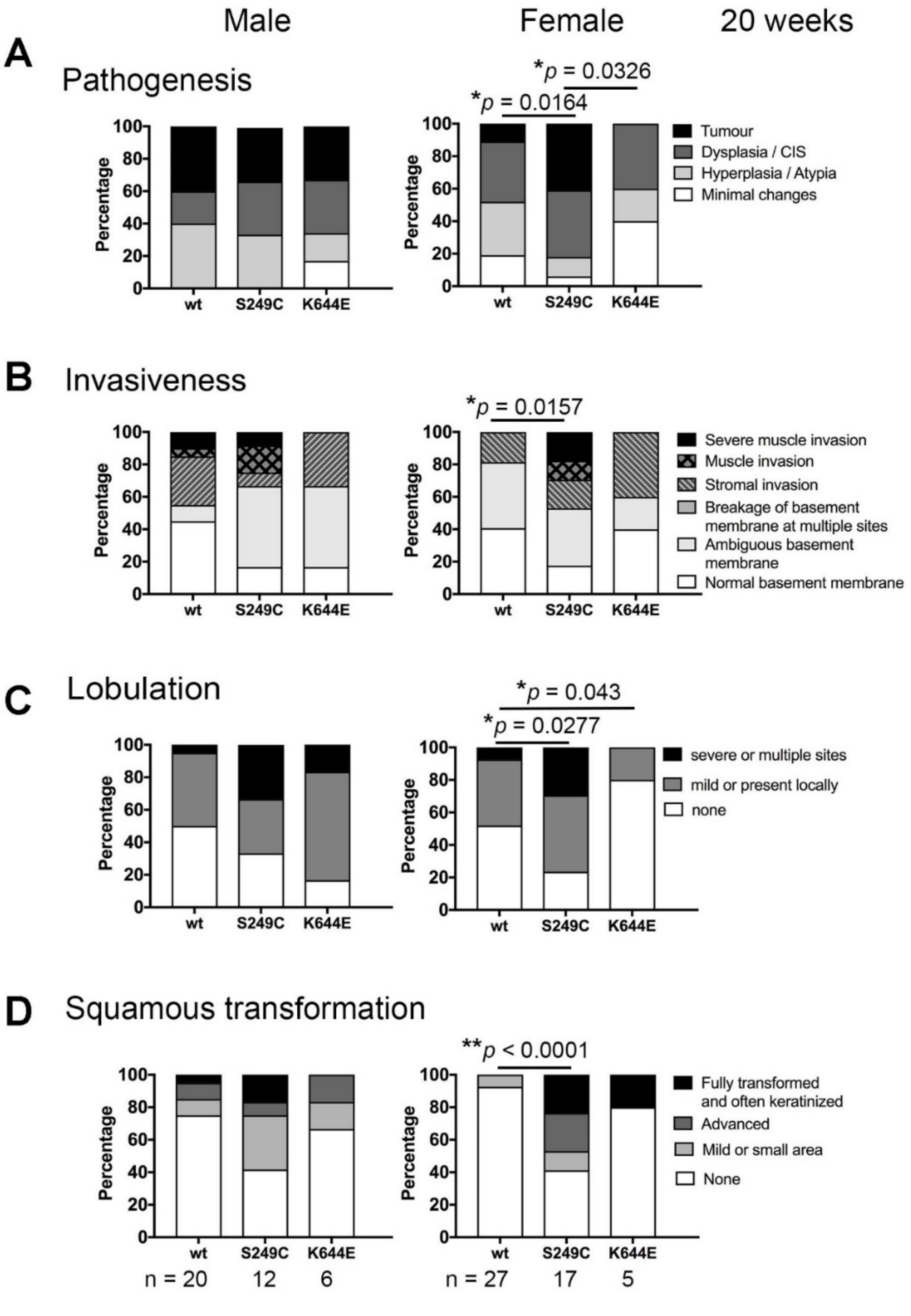

Figure S2. Histopathology of the urothelium and the bladder tumours at 20 weeks from the start of the carcinogen treatment. The phenotype was analysed in male and female cohorts separately. Frequency of the observed phenotype was shown as percentage within the samples analysed. **(A)** Pathogenesis observed in the bladder. 23.4% of *Wildtype* mice (n=11/47) formed well-recognisable tumours of an invasive nature (Fig 2I). When male and female mice were analysed separately, tumours had occurred in 40.0% (n=8/20) and 11.1% (n=3/27), respectively. This is in concordance with the increased frequency of bladder cancer in males. (**B**) Invasiveness of the urothelial and tumour cells. (**C**) Lobulated appearance of basement membrane. (**D**) Squamous differentiation observed in the urothelium and the tumour. Number of samples analysed for each phenotype is indicated below each column. The *p*-values (Mann-Whitney) are indicated above the columns, when significant (* <0.05 and ** < 0.005).

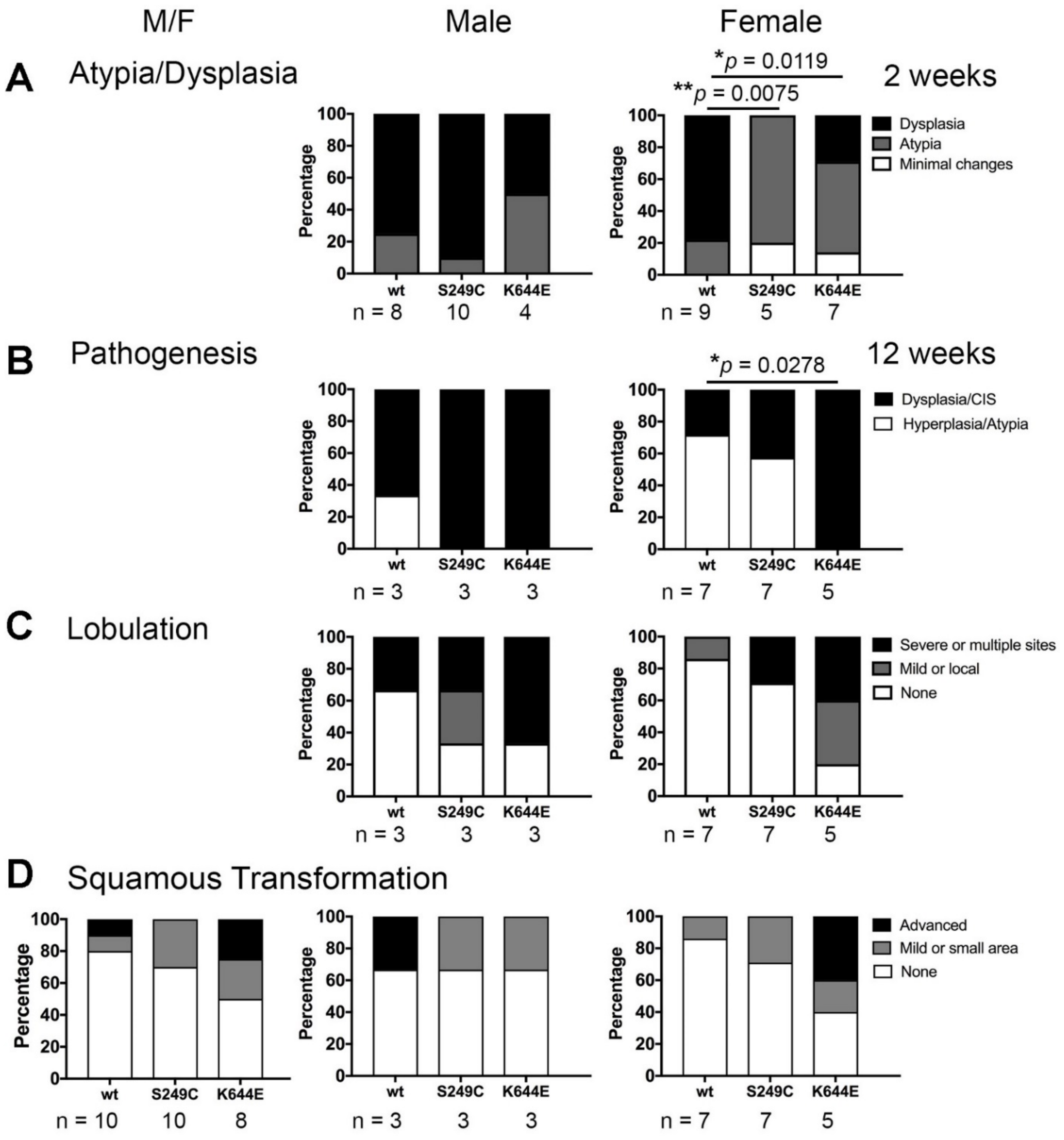

Figure S3. Histopathology of the bladder at 2 and 12 weeks from the start of the carcinogen treatment. Phenotype was analysed in both genders (**M/F**), male and female cohorts at 2 weeks (**A**) and 12 weeks (**B-D**). The frequency of each phenotypic criterion was shown as percentage within mice analysed. Presence of atypia and dysplasia (**A**) in the urothelium at 2 weeks, (**B**) Pathogenesis observed in the bladder, (**C**) lobulated appearance of the basement membrane and (**D**) squamous differentiation observed in the urothelium at 12 weeks were scored. Results of the analysis of M/F combined for atypia/dysplasia at 2 weeks, pathogenesis and lobulation at 12 weeks are presented in Figure 3. Number of samples analysed is indicated below panels. The *p*-values (Mann-Whitney) are indicated where significant (* <0.05 and ** < 0.005).

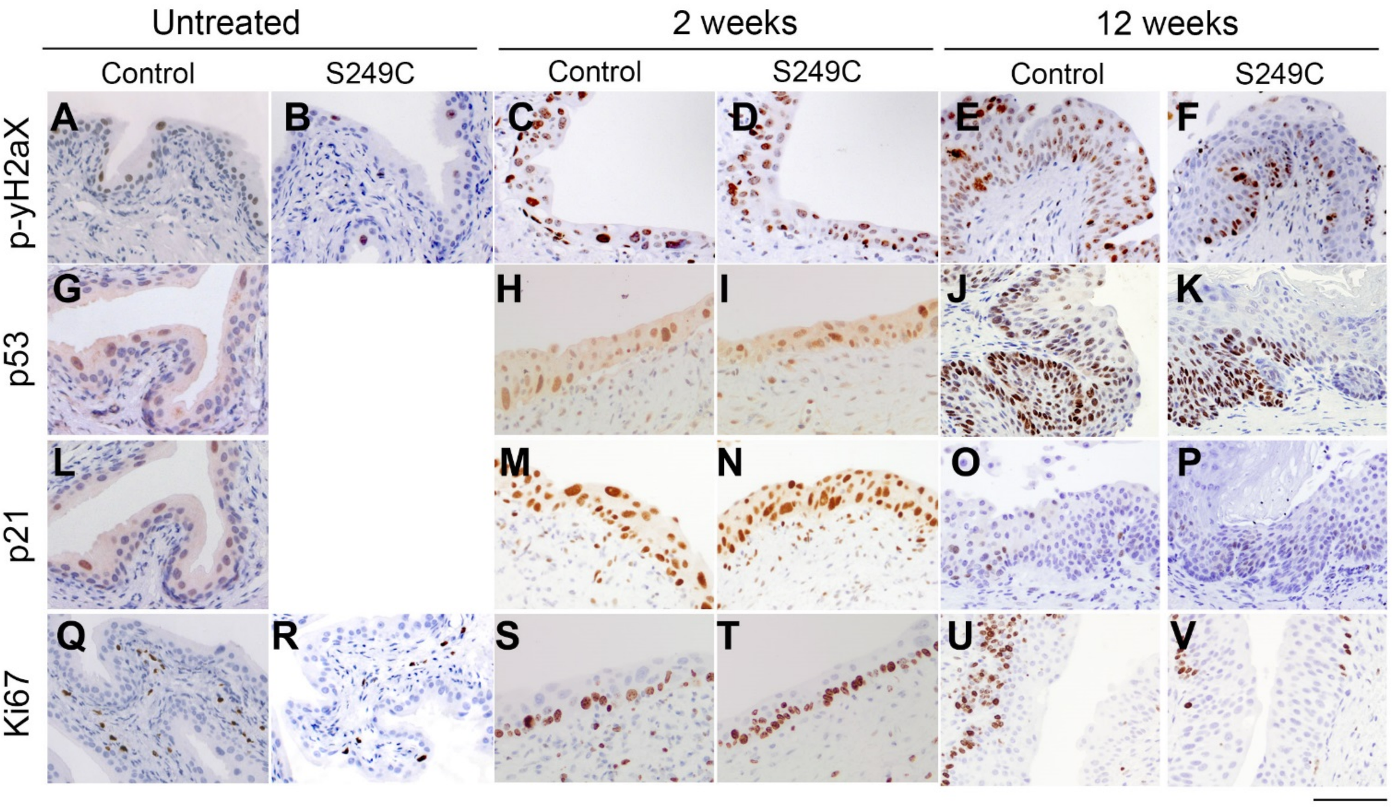

Figure S4. Response to DNA-damaging effects of OH-BBN treatment. The absence of specific differences in the pattern of γH2aX phosphorylation, expression of p53, p21, proliferation between *Wildtype* and *FGFR3*^{S249C}, excludes dysregulation of the Phospho-γH2aX-p53 axis as the potential cause of malignancy. IHC was performed in the minimum of n=5 samples per cohort at each time point. Representative sections of Wildtype and *FGFR3*^{S249C} bladders without OH-BBN treatment (**A, B, G, L, Q, R**), at 2 weeks (**C, D, H, I, M, N, S, T**) and 12 weeks from the start of the OH-BBN treatment **(E, F, J, K, O, P, U, V)** stained by Phospho-γH2aX **(**A-F), p53 (**G-K**), p21 (**L-P**) and Ki67 (**Q-V**). When untreated with OH-BBN, *FGFR3*^{S249C} bladders showed similar staining pattern to controls, including p53 and p21 (**G, L**). In the absence of carcinogen treatment, γH2aX phosphorylation was scarcely seen, indicating that the DNA damage was minimal in both cohorts (**A, B**). Proliferation of the urothelial cells was similarly low in *Wildtype* and *FGFR3*^{S249C} (**Q, R**), consistent with our earlier observations [30,31]. At 2 weeks of OH-BBN treatment, increased γH2aX phosphorylation was seen in *Wildtype* and *FGFR3*^{S249C} (**C, D**) compared to untreated urothelia (**A, B**). p53 protein levels were elevated along the basal cell layer as well as in some of the intermediate cells (**H, I**). p21 expression was evident in all urothelial layers (**M, N**). Increased proliferation was typically found along the basal layer of cells (**S, T**). These changes appeared to be according to the expected response to the carcinogen, and were similar between *Wildtype* and *FGFR3*^{S249C} cohorts. At 12 weeks, γH2aX phosphorylation generally remained in all layers of the urothelium in *Wildtype*, as well as in *FGFR3*^{S249C} mice (**E, F**). However, cells that had undergone squamous cell transformation appeared to have lost γH2aX phosphorylation in the basal cell layer (**F**), indicating that cells with DNA damage were cleared from these areas. Expression of p53 was strong in all layers of the urothelium, in particular along the basal cell layer and in some of the intermediate cells (**J, K**), while some urothelial regions lacked p53 expression (**K**). Compared to 2 weeks of OH-BBN exposure, the expression of p21 was less intense at 12 weeks (**O, P**) and no longer showed any association with regions of high p53 expression as seen at 2 weeks. Proliferation was generally observed along the basal cell layer and often regionally intense (**U, V**). Scale bar represents 100 µm in all panels.

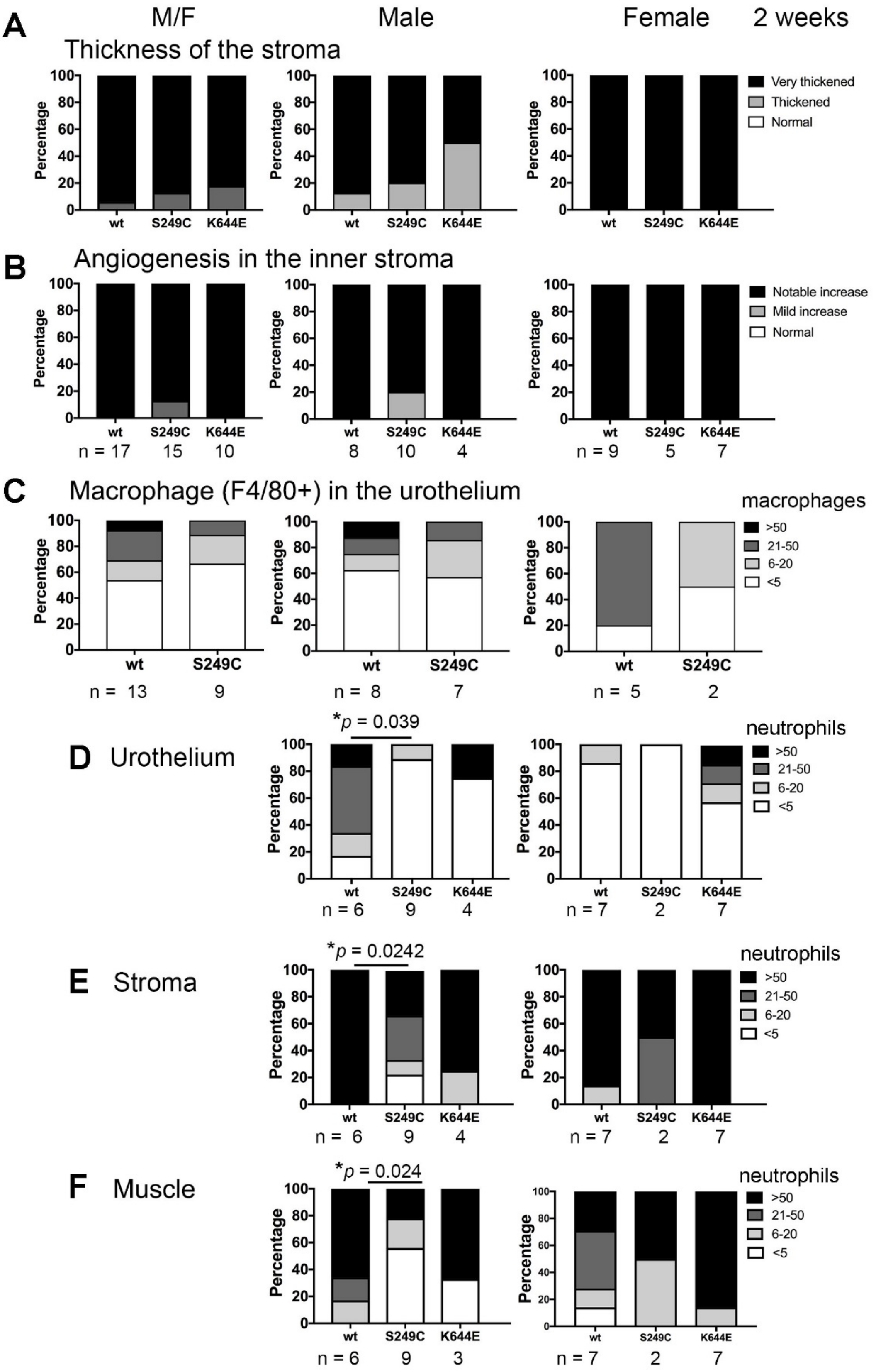

Figure S5. Inflammatory characteristics of the bladder at 2 weeks of carcinogen treatment. Phenotype was analysed in both genders (**M/F**), male and female cohorts and shown as percentage within the samples analysed. (**A**) Thickness of the stroma and (**B**) the presence of blood vessels in the stroma closer to the urothelium (inner stroma). No difference was detected in the presence of blood vessels closer to the muscle (the outer stroma) comparing cohorts (data not shown). (**C**) Macrophages in the urothelium were scored using IHC with F4/80. There were no differences in the number of macrophages in the stroma and muscle comparing *Wildtype* and *FGFR3*^{S249C} (data not shown). Presence of neutrophils in the urothelium (**D**), stroma (**E**), and muscle layer (**F**) of the bladder. Number of samples analysed is indicated below panels. Number of samples analysed is indicated below each column. The *p*-values (Mann-Whitney) are indicated where significant (* <0.05).

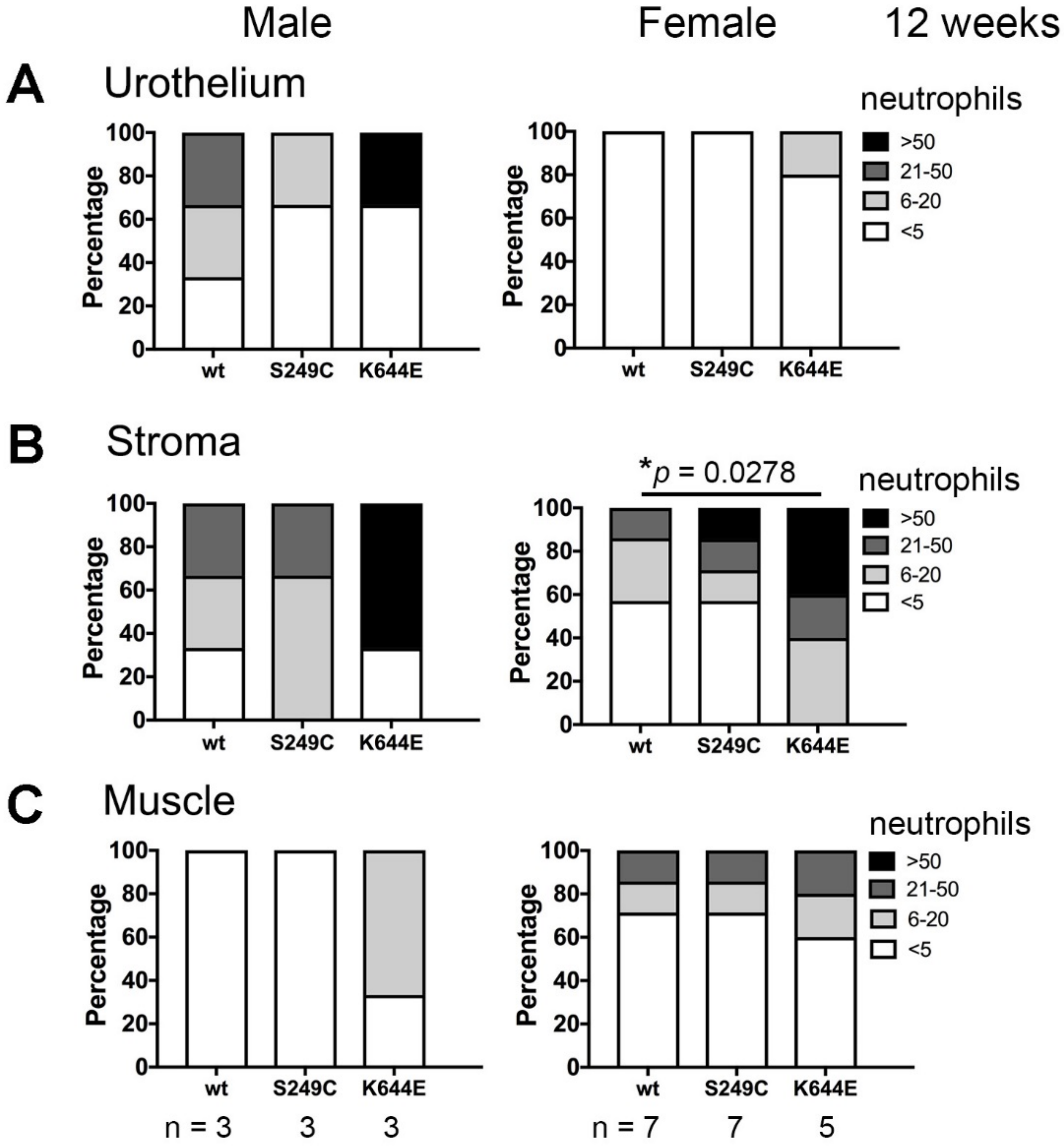

Figure S6. Presence of the neutrophils in the bladder at 12 weeks of carcinogen treatment. Presence of neutrophils in the urothelium (**A**), stroma (**B**)**,** and muscle layer (**D**) of the bladder at 12 weeks from the start of OH-BBN treatment. Phenotype was analysed in male and female cohorts and shown as percentage within the samples analysed. Number of samples analysed is indicated below each column in C. The *p*-values (Mann-Whitney) are indicated where significant (* <0.05).

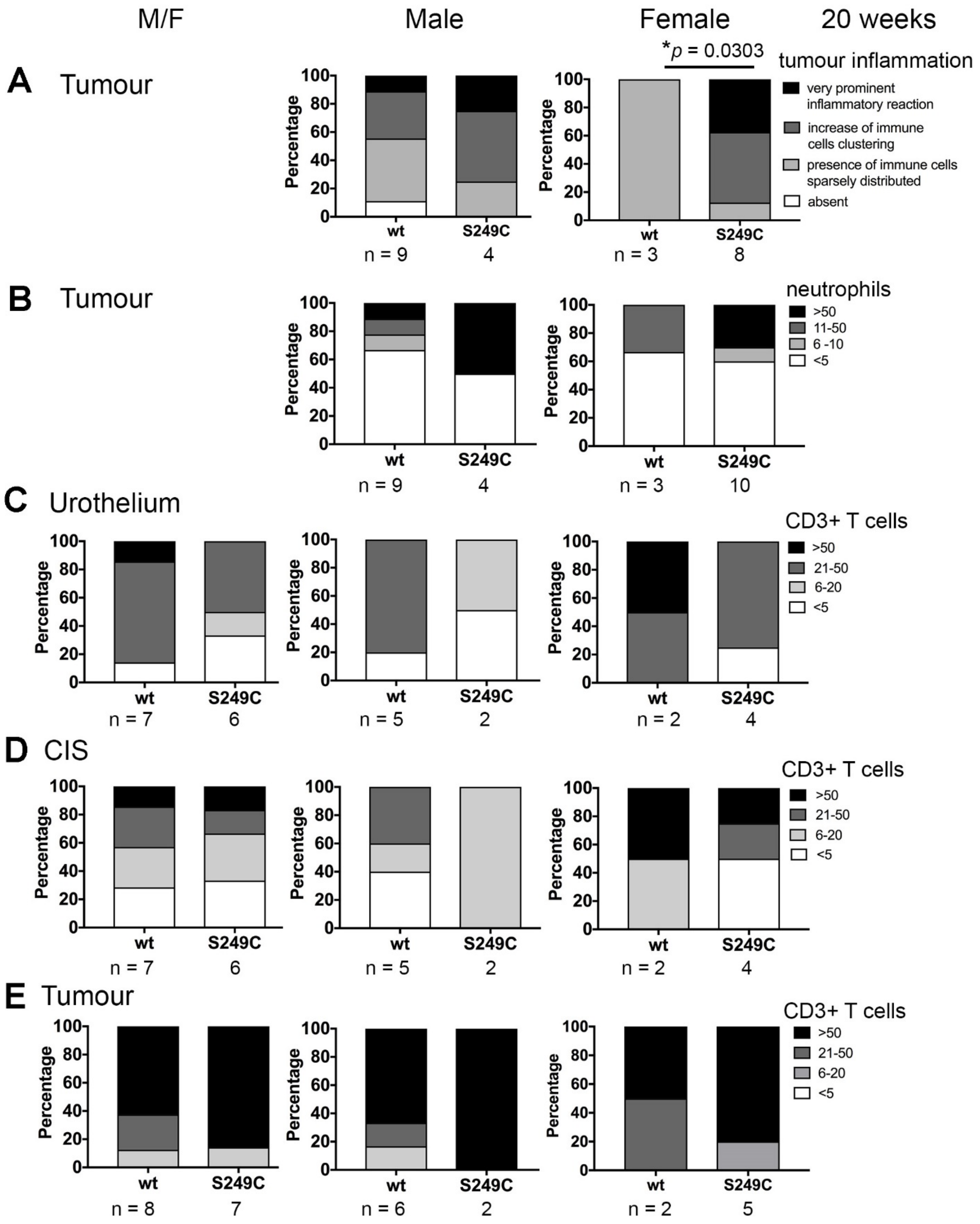

Figure S7. Inflammatory phenotype of the bladder at 20 weeks from the start of application of carcinogen. Presence of overall levels of inflammatory infiltrations (**A**) and neutrophils (**B**) in the tumour was analysed in males and females individually. Infiltration of the T-cell population was analysed using CD3 as a marker in the urothelium (**C**), CIS (**D**) and in tumours (**E**). The Y axis indicates percentage within the samples analysed. Number of samples analysed is indicated below each column. The *p*-values (Mann-Whitney) are indicated where significant (* <0.05).

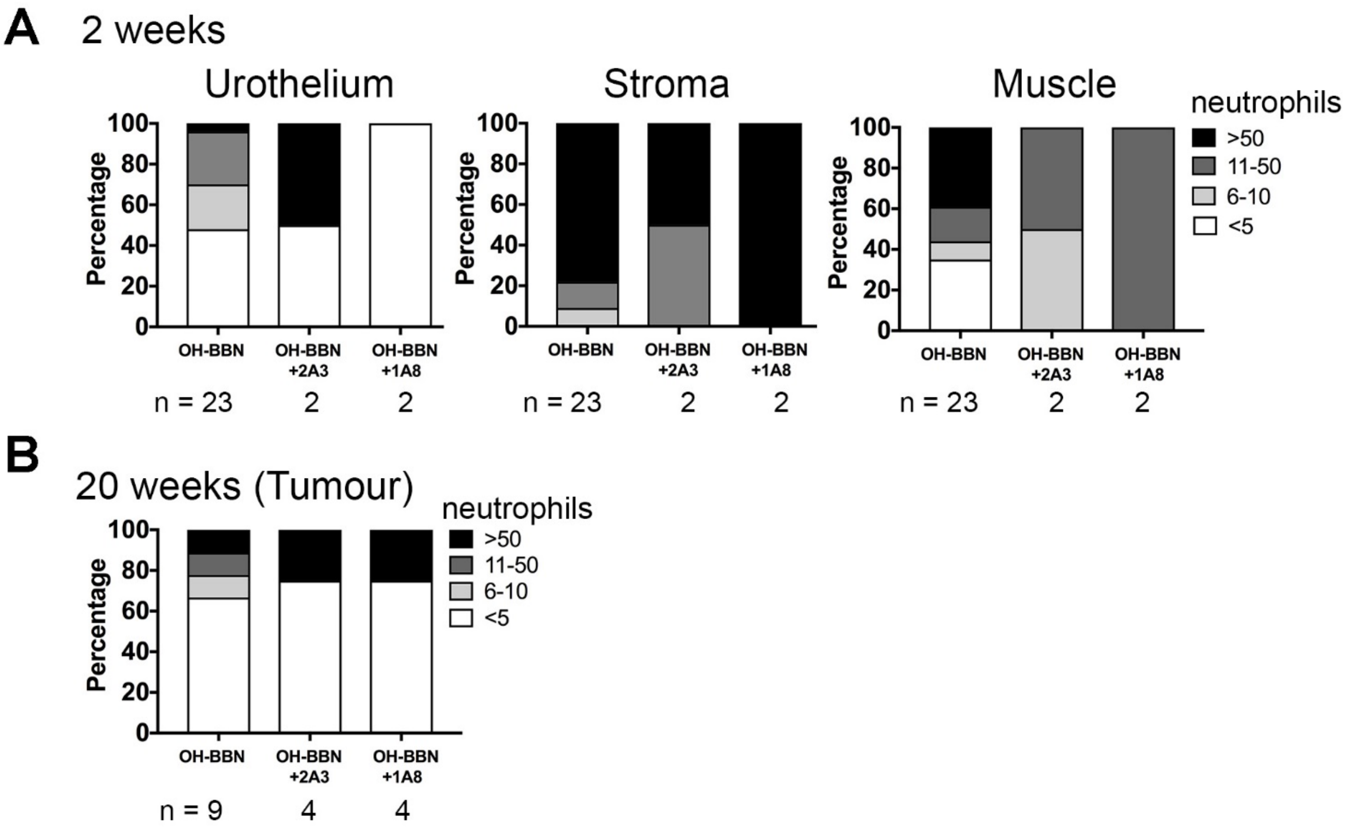

Figure S8. Presence of neutrophils in the bladder tissues upon neutrophil depletion. (**A**) Efficiency of neutrophil depletion was evaluated in the bladder tissues at 2 weeks from the start of the carcinogen treatment. (**B**) Infiltration of neutrophils in the tumour were examined at 20 weeks time point. The Y axis indicates percentage within the samples analysed. Number of samples analysed is indicated below each column. Differences were not statistically significant.

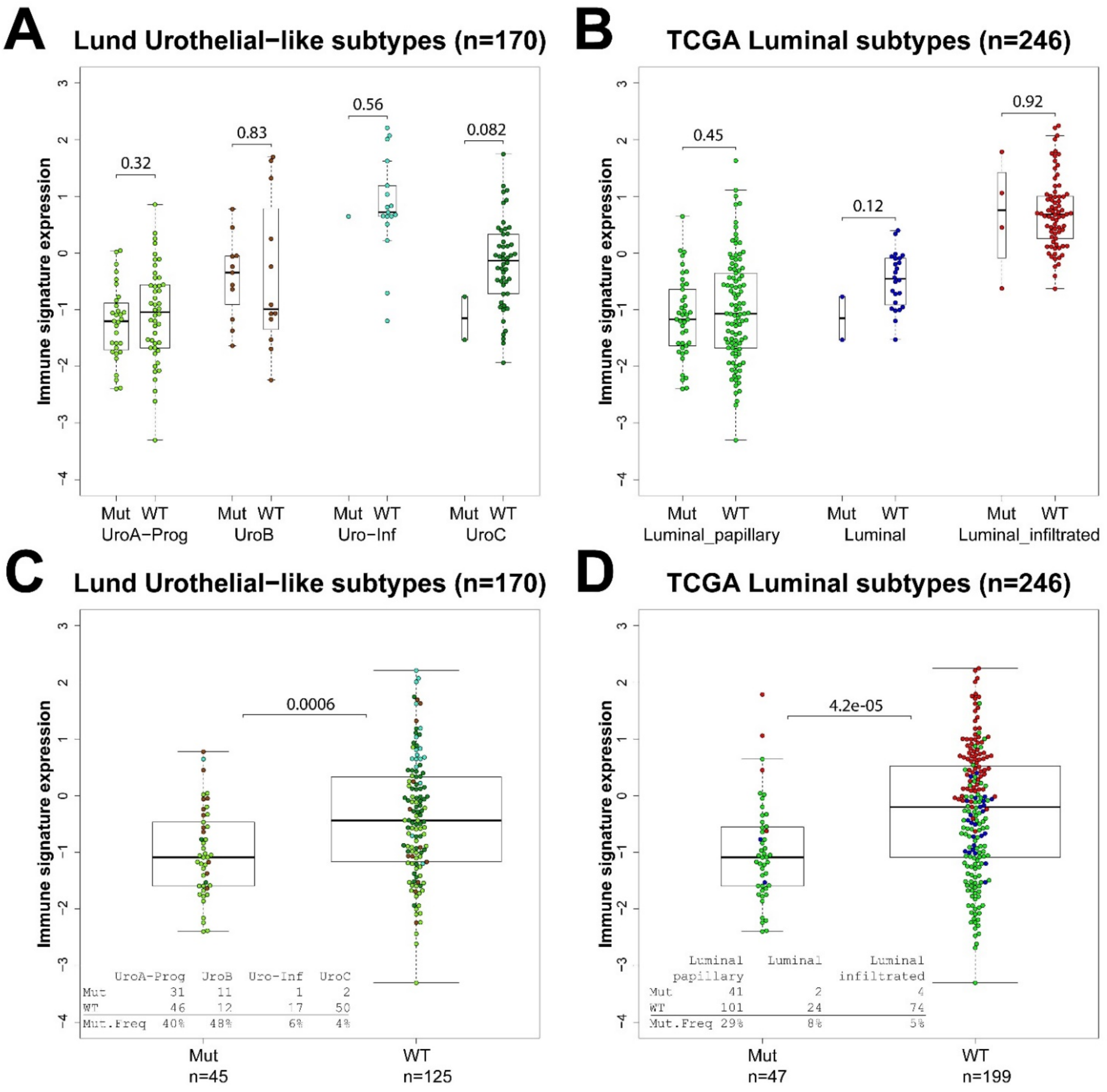

Figure S9. Immune signature expression in the TCGA dataset comparing *FGFR3* mutant and Wildtype stratified by Lund or TCGA molecular subtype. Immune infiltration signature in Lund Urothelial-Like subtype (n=170) (**A**), TCGA Luminal subtype (n=246) (**B**), and all tumours grouped by *FGFR3* mutation status in Lund Urothelial-Like subtype (**C**), and TCGA Luminal subtype **(D)**. The *p*-values (Wilcoxon rank test) are indicated. The expression of immune genes was not affected strongly by the presence or absence of *FGFR3* mutations within the different subtypes of the Lund or TCGA classification (**A, B**). The immune expression did not vary significantly within Urothelial-like B (UroB) based on *FGFR3* mutational status (p=0.83), while UroB as a whole and UroB with *FGFR3* mutations were more infiltrated than Urothelial-like A-Progressed (UroA-Prog) (p=0.0067 and 0.01 respectively) (**A**). FGFR3 mutations are uncommon in the Urothelial-like Infiltrated (Uro-Inf) and Urothelial-like C (UroC) groups. The infiltration level in Uro-Inf and UroC was higher than in UroA-Prog tumors (p= 4.8e-09 and 8.9e-09, respectively). Similarly, no difference in infiltration was seen between *FGFR3* mutated and wild-type tumors of the TCGA Luminal-Infiltrated group (p=0.92) (**B**). TCGA Luminal-Infiltrated had higher immune infiltration than the Luminal-Papillary group (p<2.2e-16). (**C, D**) When grouped by *FGFR3* mutation status, it appears that *FGFR3* mutated Urothelial-like/Luminal tumors had moderately lower immune infiltration. However, the lower infiltration seen in the mutated cases is due to an enrichment of mutations in the less infiltrated UroA-prog and Luminal-Papillary subtypes, since the mutated cases in **C, D** are not significantly different from *FGFR3* wild-type UroA-prog/Luminal-papillary tumours, as shown in **A, B**.
