# Supplementary material for: FGFR3 mutation increases bladder tumourigenesis by suppressing acute inflammation
Source: J Pathol. 2018 Sep 19;246(3):331–43. doi: 10.1002/path.5143 (PMC6334176; doi:10.1002/path.5143)
Supplement: Supplementary file 3 — Table S1. Summary of phenotype compared by gender Table S2. Correlation of inflammatory phenotype by cohort and by bladder phenotype at 2 weeks Table S3. Correlation of inflammatory phenotype by cohort and by bladder phenotype at 12 weeks Table S4. Correlation of inflammatory phenotype by cohort and by tumour phenotype at 20 weeks [file PATH-246-331-s003.pdf]

Table S1. Summary of phenotype compared by gender

Statistical significance of phenotype of each *FGFR3* mutant cohort compared to that of *Wildtype* is summarised by male and females together (M/F), males only (M) and females only (F). Where significant (<0.05), the *p*-values (Mann-Whitney) are highlighted in colour to facilitate the evaluation by gender and by genotype. "ns"; not significant.

Phenotype at 2 weeks

Figure 3 and S3	M/F			M			F		
	Wt	S249C	K644E	Wt	S249C	K644E	Wt	S249C	K644E
n	17	15	10	8	10	4	9	5	7
**Atypia/Dysplasia**		ns	0.0107		ns	ns		0.0075	0.0119

Figure 4 and S5	Wt	S249C	K644E	Wt	S249C	K644E	Wt	S249C	K644E
n	13	11	11	6	9	4	7	2	7
**neutrophils-urothelium**		0.0466	ns		0.039	ns		ns	ns
n	13	11	11	6	9	4	7	2	7
**neutrophils-stroma**		0.0063	ns		0.0242	ns		ns	ns
n	13	11	10	6	9	3	7	2	7
**neutrophils-muscle**		0.0464	ns		0.024	ns		ns	ns

Phenotype at 12 weeks

Figure 3 and S3	M/F			M			F		
	Wt	S249C	K644E	Wt	S249C	K644E	Wt	S249C	K644E
n	10	10	8	3	3	3	7	7	5
**Pathogenesis**		ns	0.0128		ns	ns		ns	0.0278
**Lobulation**		ns	0.0296		ns	ns		ns	ns

Figure 4 and S6	Wt	S249C	K644E	Wt	S249C	K644E	Wt	S249C	K644E
n	10	10	8	3	3	3	7	7	5
**neutrophils-urothelium**		ns	ns		ns	ns		ns	ns
**neutrophils-stroma**		ns	0.0229		ns	ns		ns	0.0278
**neutrophils-muscle**		ns	ns		ns	ns		ns	ns

Phenotype at 20 weeks

Figure 2 and S2	M/F			M			F		
	Wt	S249C	K644E	Wt	S249C	K644E	Wt	S249C	K644E
n	47	29	11	20	12	6	27	17	5
**Pathogenesis**		0.0454	ns		ns	ns		0.0164	ns
**Invasiveness**		0.0239	ns		ns	ns		0.0157	ns
**Lobulation**		0.0073	ns		ns	ns		0.0277	0.043
**Squamous transformation**		<0.0001	ns		ns	ns		<0.0001	ns

Figure 4 and S7	Wt	S249C		Wt	S249C		Wt	S249C
n	12	12		9	4		3	8
**tumour inflammation**		0.018			ns			0.0303
**tumour infiltrating neutrophils**		ns			ns			ns

Table S2. Correlation of inflammatory phenotype by cohort and by bladder phenotype at 2 weeks

Spearman's rho correlation coefficients were evaluated across cohorts and scoring criteria (Table S1.1 to S1.3) and the Kruskal-Wallis test was performed, grouped by Cohort (Table S2.4 to S2.6), and by Dysplasia (Table S2.7 to S2.9). *Red and ****bolded** red figures indicate that the correlation is significant at the 0.05 and 0.01 level (2-tailed), respectively.

Table S2.1 Spearman rank correlation (M/F) of cohort and all phenotype at 2 weeks.

	Cohort	Hyperplasia	Dysplasia	Thickness of the stroma	Angiogenesis in the inner stroma	Neutrophils (urothelium)	Neutrophils (stroma)	Neutrophils (muscle)
n	43	43	43	43	43	33	33	32
Cohort	1.000							
Hyperplasia	0.070	1.000						
Dysplasia	**-0.405****	0.075	1.000					
Thickness of the stroma	-0.156	-0.058	-0.168	1.000				
Angiogenesis in the inner stroma	-0.057	0.089	-0.184	-0.080	1.000			
Neutrophils (urothelium)	-0.059	0.064	-0.269	0.111	0.122	1.000		
Neutrophils (stroma)	-0.089	0.255	-0.127	-0.096	.0308	0.417*	1.000	
Neutrophils (muscle)	0.054	0.040	-0.336	-0.070	0.259	**0.531****	**0.803****	1.000

Table S2.2 Spearman rank correlation (Male) of cohort and all phenotype at 2 weeks

	Cohort	Hyperplasia	Dysplasia	Thickness of the stroma	Angiogenesis in the inner stroma	Neutrophils (urothelium)	Neutrophils (stroma)	Neutrophils (muscle)
n	22	22	22	22	22	18	18	18
Cohort	1.000							
Hyperplasia	-0.184	1.000						
Dysplasia	-0.111	-0.064	1.000					
Thickness of the stroma	-0.278	-0.013	-0.035	1.000				
Angiogenesis in the inner stroma	-0.108	0.149	-0.171	-0.171	1.000			
Neutrophils (urothelium)	**-0.760****	0.383	0.216	0.106	0.148	1.000		
Neutrophils (stroma)	-0.304	0.345	-0.272	-0.287	0.320	0.471*	1.000	
Neutrophils (muscle)	-0.187	0.123	-0.365	-0.192	0.300	0.543*	**0.874****	1.000

Table S2.3 Spearman rank correlation (Female) of cohort and all phenotype at 2 weeks

	Cohort	Hyperplasia	Dysplasia	Neutrophils (urothelium)	Neutrophils (stroma)	Neutrophils (muscle)
n	21	21	21	15	15	14
Cohort	1.000					
Hyperplasia	0.401	1.000				
Dysplasia	**-0.627****	0.117	1.000			
Neutrophils (urothelium)	0.565*	-0.309	-0.593*	1.000		
Neutrophils (stroma)	0.188	0.153	0.304	0.308	1.000	
Neutrophils (muscle)	0.372	-0.055	-0.289	0.514	0.607*	1.000

Table S2.4 Kruskal-Wallis test (M/F) of phenotype grouped by cohort at 2 weeks

	Hyperplasia	Dysplasia	Thickness of the stroma	Angiogenesis in the inner stroma	Neutrophils (urothelium)	Neutrophils (stroma)	Neutrophils (muscle)
Chi-Square	0.213	7.360	1.024	3.824	3.881	11.760	8.132
df	2	2	2	2	2	2	2
Asymp. Sig.	0.899	0.025	0.599	0.148	0.144	**0.003**	0.017

Table S2.5 Kruskal-Wallis test (Male) of phenotype grouped by cohort at 2 weeks

	Hyperplasia	Dysplasia	Thickness of the stroma	Angiogenesis in the inner stroma	Neutrophils (urothelium)	Neutrophils (stroma)	Neutrophils (muscle)
Chi-Square	1.054	2.520	2.112	2.520	12.729	7.315	7.170
df	2	2	2	2	2	2	2
Asymp. Sig.	0.590	0.284	0.348	0.284	**0.002**	0.026	0.028

Table S2.6 Kruskal-Wallis test (Female) of phenotype grouped by cohort at 2 weeks

	Hyperplasia	Dysplasia	Neutrophils (urothelium)	Neutrophils (stroma)	Neutrophils (muscle)
Chi-Square	4.211	9.961	4.483	2.663	1.920
df	2	2	2	2	2
Asymp. Sig.	0.122	**0.007**	0.106	0.264	0.383

Table S2.7 Kruskal-Wallis test (M/F) of phenotype by dysplasia at 2 weeks

	Cohort	Hyperplasia	Thickness of the stroma	Angiogenesis in the inner stroma	Neutrophils (urothelium)	Neutrophils (stroma)	Neutrophils (muscle)
Chi-Square	7.167	0.531	1.191	1.475	4.329	0.648	3.586
df	2	2	2	2	2	2	2
Asymp. Sig.	0.028	0.767	0.551	0.478	0.115	0.723	0.166

Table S2.8 Kruskal-Wallis test (Male) of phenotype grouped by dysplasia at 2 weeks

	Cohort	Hyperplasia	Thickness of the stroma	Angiogenesis in the inner stroma	Neutrophils (urothelium)	Neutrophils (stroma)	Neutrophils (muscle)
Chi-Square	0.259	0.085	0.026	0.618	0.796	1.261	2.261
df	1	1	1	1	1	1	1
Asymp. Sig.	0.611	0.770	0.872	0.432	0.372	0.261	0.133

Table S2.9 Kruskal-Wallis test (Female) of phenotype grouped by dysplasia at 2 weeks

	Cohort	Hyperplasia	Thickness of the stroma	Angiogenesis in the inner stroma	Neutrophils (urothelium)	Neutrophils (stroma)	Neutrophils (muscle)
Chi-Square	7.980	0.382	0.000	0.000	5.453	2.449	1.316
df	2	2	2	2	2	2	2
Asymp. Sig.	0.019	0.826	1.000	1.000	0.065	0.294	0.518

Table S3. Correlation of inflammatory phenotype by cohort and by bladder phenotype at 12 weeks

Spearman's rho correlation coefficients were evaluated across cohort and scoring criteria (Table S3.1 to S3.3) and the Kruskal-Wallis test was performed, grouped by Cohort (Table S3.4 to S3.6) and Pathogenesis (Table 3.7 to Table 3.9). *Red and ****bolded** red figures indicate that the correlation is significant at the 0.05 and 0.01 level (2-tailed), respectively.

Table S3.1 Spearman rank correlation (M/F) of cohort and all phenotype at 12 weeks

	Cohort	Pathogenesis	Lobulation	Squamous transformation	Neutrophils (urothelium)	Neutrophils (stroma)	Neutrophils (muscle)
N	29	28	28	28	28	28	28
Cohort	1.000						
Pathogenesis	0.260	1.000					
Lobulation	0.435*	**0.621****	1.000				
Squamous transformation	0.256	**0.707****	**0.832****	1.000			
Neutrophils (urothelium)	0.044	-0.038	0.190	-0.060	1.000		
Neutrophils (stroma)	0.431*	0.356	**0.671****	**0.508****	**0.441***	1.000	
Neutrophils (muscle)	0.226	0.174	0.246	0.012	0.139	**0.596****	1.000

Table S3.2 Spearman rank correlation (Male) of cohort and all phenotype at 12 weeks

	Cohort	Pathogenesis	Lobulation	Squamous transformation	Neutrophils (urothelium)	Neutrophils (stroma)	Neutrophils (muscle)
N	9	9	9	9	9	9	9
Cohort	1.000						
Pathogenesis	0.000	1.000					
Lobulation	0.289	0.567	1.000				
Squamous transformation	-0.094	**0.804****	0.736*	1.000			
Neutrophils (urothelium)	-0.145	0.114	0.377	0.011	1.000		
Neutrophils (stroma)	0.353	0.533	0.776*	0.375	0.572	1.000	
Neutrophils (muscle)	0.655	0.357	0.567	0.124	0.228	0.747*	1.000

Table S3.3 Spearman rank correlation (Female) of cohort and all phenotype at 12 weeks

	Cohort	Pathogenesis	Lobulation	Squamous transformation	Neutrophils (urothelium)	Neutrophils (stroma)	Neutrophils (muscle)
N	20	19	19	19	19	19	19
Cohort	1.000						
Pathogenesis	0.395	1.000					
Lobulation	0.513*	**0.674****	1.000				
Squamous transformation	0.425	**0.666****	**0.918****	1.000			
Neutrophils (urothelium)	0.320	-0.141	-0.175	-0.158	1.000		
Neutrophils (stroma)	0.477*	0.310	**0.611****	**0.577****	0.363	1.000	
Neutrophils (muscle)	0.086	0.106	0.173	-0.025	0.419	**0.619****	1.000

Table S3.4 Kruskal-Wallis test (M/F) of phenotype grouped by cohort at 12 weeks

	Pathogenesis	Lobulation	Squamous transformation	Neutrophils (urothelium)	Neutrophils (stroma)	Neutrophils (muscle)
Chi-Square	1.929	5.213	2.090	0.844	5.651	1.989
df	2	2	2	2	2	2
Asymp. Sig.	0.381	0.074	0.352	0.656	0.059	0.370

Table S3.5 Kruskal-Wallis test (Male) of phenotype grouped by cohort at 12 weeks

	Pathogenesis	Lobulation	Squamous transformation	Neutrophils (urothelium)	Neutrophils (stroma)	Neutrophils (muscle)
Chi-Square	1.143	0.667	0.095	0.673	1.015	4.571
df	2	2	2	2	2	2
Asymp. Sig.	0.565	0.717	0.953	0.714	0.602	0.102

Table S3.6 Kruskal-Wallis test (Female) of phenotype grouped by cohort at 12 weeks

	Pathogenesis	Lobulation	Squamous transformation	Neutrophils (urothelium)	Neutrophils (stroma)	Neutrophils (muscle)
Chi-Square	3.762	5.067	3.720	2.800	5.151	0.204
df	2	2	2	2	2	2
Asymp. Sig.	0.152	0.079	0.156	0.247	0.076	0.903

Table S3.7 Kruskal-Wallis test (M/F) of phenotype by pathogenesis at 12 weeks

	Cohort	Lobulation	Squamous transformation	Neutrophils (urothelium)	Neutrophils (stroma)	Neutrophils (muscle)
Chi-Square	0.821	12.555	16.823	0.179	1.707	2.095
df	1	1	1	1	1	1
Asymp. Sig.	0.365	**0.000**	**0.000**	0.673	0.191	0.148

Table S3.8 Kruskal-Wallis test (Male) of phenotype grouped by pathogenesis at 12 weeks

	Cohort	Lobulation	Squamous transformation	Neutrophils (urothelium)	Neutrophils (stroma)	Neutrophils (muscle)
Chi-Square	0.000	2.571	5.173	0.104	2.276	1.020
df	1	1	1	1	1	1
Asymp. Sig.	1.000	0.109	0.023	0.747	0.131	0.312

Table S3.9 Kruskal-Wallis test (Female) of phenotype grouped by pathogenesis at 12 weeks

	Cohort	Lobulation	Squamous transformation	Neutrophils (urothelium)	Neutrophils (stroma)	Neutrophils (muscle)
Chi-Square	2.802	8.169	7.983	0.357	1.732	0.204
df	1	1	1	1	1	1
Asymp. Sig.	0.094	**0.004**	**0.005**	0.550	0.188	0.652

Table S4. Correlation of inflammatory phenotype by cohort and by tumour phenotype at 20 weeks

Spearman's rho correlation coefficients were evaluated across cohort and scoring criteria (Table S4.1 to S4.3) and the Kruskal-Wallis test was performed grouped by Cohort (Table S4.4 to S4.6), Pathogenesis (Table S4.7 to S4.9), and Invasiveness (Table S4.10 to S4.12). *Red and ****bolded** red figures indicate that the correlation is significant at the 0.05 and 0.01 level (2-tailed), respectively.

Table S4.1 Spearman rank correlation (M/F) of cohort and all phenotype at 20 weeks

	Cohort	Pathogenesis	Invasiveness	Lobulation	Squamous transformation	Inflammation (urothelium)	Inflammation (stroma)	Inflammation (muscle)	Inflammation (tumour)
N	87	87	87	87	87	87	87	87	87
Cohort	1.000								
Pathogenesis	0.077	1.000							
Invasiveness	0.188	**0.889****	1.000						
Lobulation	0.197	**0.787****	**0.717****	1.000					
Squamous transformation	**0.323****	**0.623****	**0.628****	**0.676****	1.000				
Inflammation (urothelium)	0.113	0.127	0.098	0.176	0.062	1.000			
Inflammation (stroma)	-0.055	0.190	0.185	0.195	0.144	0.187	1.000		
Inflammation (muscle)	-0.109	**0.324****	**0.310****	0.252*	**0.290****	**0.315****	**0.328****	1.000	
Inflammation (tumour)	0.117	**0.753****	**0.763****	**0.521****	**0.623****	-0.027	0.142	0.248*	1.000

Table S4.2 Spearman rank correlation (Male) of cohort and all phenotype at 20 weeks

	Cohort	Pathogenesis	Invasiveness	Lobulation	Squamous transformation	Inflammation (urothelium)	Inflammation (stroma)	Inflammation (muscle)	Inflammation (tumour)
N	38	38	38	38	38	38	38	38	38
Cohort	1.000								
Pathogenesis	-0.060	1.000							
Invasiveness	0.077	**0.912****	1.000						
Lobulation	0.299	**0.675****	**0.602****	1.000					
Squamous transformation	0.177	**0.625****	**0.646****	**0.713****	1.000				
Inflammation (urothelium)	0.089	-0.141	-0.062	0.130	0.013	1.000			
Inflammation (stroma)	-0.253	0.209	0.168	0.194	0.191	0.204	1.000		
Inflammation (muscle)	-0.303	0.229	0.186	0.091	0.256	0.171	0.386*	1.000	
Inflammation (tumour)	-0.033	**0.826****	**0.836****	**0.533****	**0.717****	-0.065	0.160	0.173	1.000

Table S4.3 Spearman rank correlation (Female) of cohort and all phenotype at 20 weeks

	Cohort	Pathogenesis	Invasiveness	Lobulation	Squamous transformation	Inflammation (urothelium)	Inflammation (stroma)	Inflammation (muscle)	Inflammation (tumour)
N	49	49	49	49	49	49	49	49	49
Cohort	1.000								
Pathogenesis	0.179	1.000							
Invasiveness	0.270	**0.875****	1.000						
Lobulation	0.114	**0.877****	**0.803****	1.000					
Squamous transformation	**0.435****	**0.613****	**0.611****	**0.641****	1.000				
Inflammation (urothelium)	0.121	0.304*	0.232	0.206	0.078	1.000			
Inflammation (stroma)	0.121	0.197	0.206	0.208	0.107	0.186	1.000		
Inflammation (muscle)	0.062	**0.375****	**0.448****	**0.406****	0.320*	**0.418****	0.323*	1.000	
Inflammation (tumour)	0.248	**0.671****	**0.680****	**0.505****	**0.561****	-0.016	0.131	0.302*	1.000

Table S4.4 Kruskal-Wallis test (M/F) of phenotype grouped by cohort at 20 weeks

	Pathogenesis	Invasiveness	Lobulation	Squamous transformation	Inflammation (urothelium)	Inflammation (stroma)	Inflammation (muscle)	Inflammation (tumour)
Chi-Square	4.551	5.222	7.505	16.883	4.304	0.784	1.199	4.866
df	2	2	2	2	2	2	2	2
Asymp. Sig.	0.103	0.073	0.023	**0.000**	0.116	0.676	0.549	0.088

Table S4.5 Kruskal-Wallis test (Male) of phenotype grouped by cohort at 20 weeks

	Pathogenesis	Invasiveness	Lobulation	Squamous transformation	Inflammation (urothelium)	Inflammation (stroma)	Inflammation (muscle)	Inflammation (tumour)
Chi-Square	0.146	0.456	3.481	3.141	0.759	2.792	3.919	0.042
df	2	2	2	2	2	2	2	2
Asymp. Sig.	0.929	0.796	0.175	0.208	0.684	0.248	0.141	0.979

Table S4.6 Kruskal-Wallis test (Female) of phenotype grouped by cohort at 20 weeks

	Pathogenesis	Invasiveness	Lobulation	Squamous transformation	Inflammation (urothelium)	Inflammation (stroma)	Inflammation (muscle)	Inflammation (tumour)
Chi-Square	8.722	5.791	7.656	14.884	3.930	0.708	0.185	10.790
df	2	2	2	2	2	2	2	2
Asymp. Sig.	0.013	0.055	0.022	**0.001**	0.140	0.702	0.912	**0.005**

Table S4.7 Kruskal-Wallis test (M/F) of phenotype grouped by pathogenesis at 20 weeks

	Cohort	Invasiveness	Lobulation	Squamous transformation	Inflammation (urothelium)	Inflammation (stroma)	Inflammation (muscle)	Inflammation (tumour)
Chi-Square	2.776	68.849	57.208	35.528	3.205	5.034	9.733	66.932
df	3	3	3	3	3	3	3	3
Asymp. Sig.	0.428	**0.000**	**0.000**	**0.000**	0.361	0.169	0.021	**0.000**

Table S4.8 Kruskal-Wallis test (Male) of phenotype grouped by pathogenesis at 20 weeks

	Cohort	Invasiveness	Lobulation	Squamous transformation	Inflammation (urothelium)	Inflammation (stroma)	Inflammation (muscle)	Inflammation (tumour)
Chi-Square	4.612	31.442	18.560	14.644	1.117	2.938	2.614	31.161
df	3	3	3	3	3	3	3	3
Asymp. Sig.	0.202	**0.000**	**0.000**	**0.002**	0.773	0.401	0.455	**0.000**

Table S4.9 Kruskal-Wallis test (Female) of phenotype grouped by pathogenesis at 20 weeks

	Cohort	Invasiveness	Lobulation	Squamous transformation	Inflammation (urothelium)	Inflammation (stroma)	Inflammation (muscle)	Inflammation (tumour)
Chi-Square	2.802	37.014	39.482	21.178	4.816	2.912	6.790	33.337
df	3	3	3	3	3	3	3	3
Asymp. Sig.	0.423	**0.000**	**0.000**	**0.000**	0.186	0.405	0.079	**0.000**

Table S4.10 Kruskal-Wallis test (M/F) of phenotype grouped by invasiveness at 20 weeks

	Cohort	Invasiveness	Lobulation	Squamous transformation	Inflammation (urothelium)	Inflammation (stroma)	Inflammation (muscle)	Inflammation (tumour)
Chi-Square	2.802	37.014	39.482	21.178	4.816	2.912	6.790	33.337
df	3	3	3	3	3	3	3	3
Asymp. Sig.	0.423	**0.000**	**0.000**	**0.000**	0.186	0.405	0.079	**0.000**

Table S4.11 Kruskal-Wallis test (Male) of phenotype grouped by invasiveness at 20 weeks

	Cohort	Pathogenesis	Lobulation	Squamous transformation	Inflammation (urothelium)	Inflammation (stroma)	Inflammation (muscle)	Inflammation (tumour)
Chi-Square	7.527	33.045	15.242	15.809	3.036	1.536	1.371	31.857
df	4	4	4	4	4	4	4	4
Asymp. Sig.	0.111	**0.000**	**0.004**	**0.003**	0.552	0.820	0.849	**0.000**

Table S4.12 Kruskal-Wallis test (Female) of phenotype grouped by invasiveness at 20 weeks

	Cohort	Pathogenesis	Lobulation	Squamous transformation	Inflammation (urothelium)	Inflammation (stroma)	Inflammation (muscle)	Inflammation (tumour)
Chi-Square	5.175	37.464	31.266	20.900	11.844	4.674	17.785	32.116
df	4	4	4	4	4	4	4	4
Asymp. Sig.	0.270	**0.000**	**0.000**	**0.000**	**0.019**	0.322	**0.001**	**0.000**
